# Interplay between chromosomal architecture and termination of DNA replication in bacteria

**DOI:** 10.3389/fmicb.2023.1180848

**Published:** 2023-06-26

**Authors:** Daniel J. Goodall, Dominika Warecka, Michelle Hawkins, Christian J. Rudolph

**Affiliations:** ^1^Division of Biosciences, College of Health, Medicine and Life Sciences, Brunel University London, Uxbridge, United Kingdom; ^2^Department of Biology, University of York, York, United Kingdom

**Keywords:** termination of DNA replication, DNA replication, DNA segregation, RecG helicase, Tus-*ter* complexes, bacterial chromosome dynamics, chromosomal architecture

## Abstract

Faithful transmission of the genome from one generation to the next is key to life in all cellular organisms. In the majority of bacteria, the genome is comprised of a single circular chromosome that is normally replicated from a single origin, though additional genetic information may be encoded within much smaller extrachromosomal elements called plasmids. By contrast, the genome of a eukaryote is distributed across multiple linear chromosomes, each of which is replicated from multiple origins. The genomes of archaeal species are circular, but are predominantly replicated from multiple origins. In all three cases, replication is bidirectional and terminates when converging replication fork complexes merge and ‘fuse’ as replication of the chromosomal DNA is completed. While the mechanics of replication initiation are quite well understood, exactly what happens during termination is far from clear, although studies in bacterial and eukaryotic models over recent years have started to provide some insight. Bacterial models with a circular chromosome and a single bidirectional origin offer the distinct advantage that there is normally just one fusion event between two replication fork complexes as synthesis terminates. Moreover, whereas termination of replication appears to happen in many bacteria wherever forks happen to meet, termination in some bacterial species, including the well-studied bacteria *Escherichia coli* and *Bacillus subtilis*, is more restrictive and confined to a ‘replication fork trap’ region, making termination even more tractable. This region is defined by multiple genomic terminator (*ter*) sites, which, if bound by specific terminator proteins, form unidirectional fork barriers. In this review we discuss a range of experimental results highlighting how the fork fusion process can trigger significant pathologies that interfere with the successful conclusion of DNA replication, how these pathologies might be resolved in bacteria without a fork trap system and how the acquisition of a fork trap might have provided an alternative and cleaner solution, thus explaining why in bacterial species that have acquired a fork trap system, this system is remarkably well maintained. Finally, we consider how eukaryotic cells can cope with a much-increased number of termination events.

## Introduction

Bacteria show a considerable degree of variability in terms of genome size and organization. Genomes can be as small as 0.159 Mbp, such as in the symbiotic bacterium *Carsonella ruddii*, which carries only 182 genes (Nakabachi et al., 2006), and as large as 13 Mbp in the myxobacterium *Sorangium cellulosum*, with 9,376 predicted open reading frames (Schneiker et al., 2007). The majority of bacteria carry a single chromosome, in the form of a covalently closed circle. About 5% carry multiple chromosomes (Touchon and Rocha, 2016). These species include *Vibrio cholerae* and close relatives, with two (Touchon and Rocha, 2016) and *Paracoccus denitrificans*, with three (Winterstein and Ludwig, 1998). A few species in the *Actinomycetales*, including the genus *Streptomyces* (Kirby, 2011), have linear chromosomes or a mixed state. For instance, *Agrobacterium tumefaciens* has one circular and one linear chromosome, along with two mega-plasmids (Nester, 2015).

**Figure 1:**
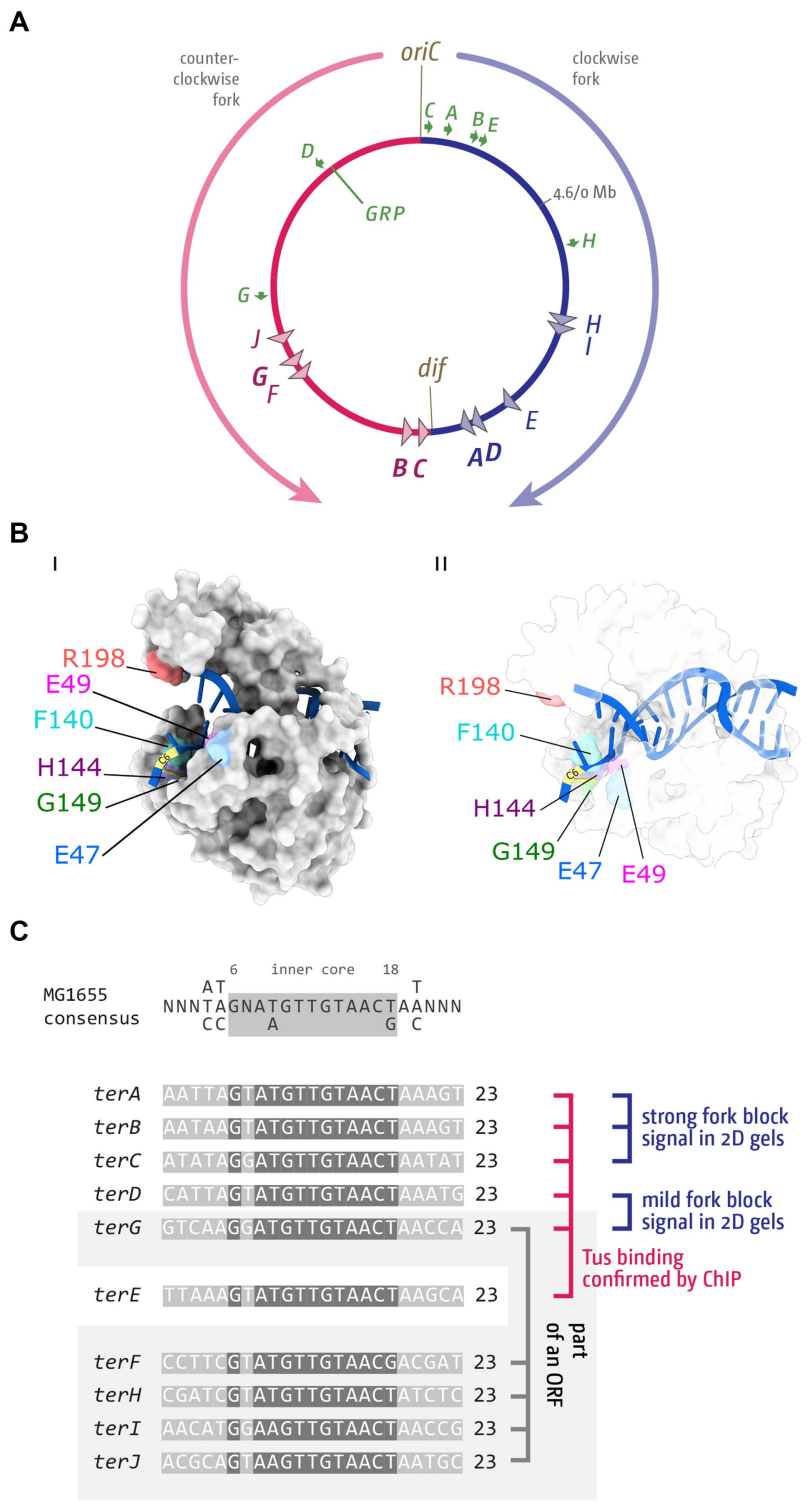
Chromosome structure and Tus-*ter* trap in *Escherichia coli*. **(A)** Schematic representation of the *E. coli* chromosome. Two replication forks are initiated at a single origin termed *oriC* and move in opposite directions along the DNA until they approach one another and fuse within the terminus region opposite *oriC*. A replication fork trap is formed in the terminus region *via* terminator sequences (*terA–J*) which are arranged as two opposed groups, with the pink terminators oriented to block movement of the clockwise replication fork and the blue terminators oriented to block the counter-clockwise fork. The locations of the *dif* chromosome dimer resolution site is marked. Locations of the *rrn* operons, which are particularly highly transcribed under fast growth conditions, are shown by green arrows, with the arrow pointing in the direction in which transcribing RNA polymerase molecules travel. ‘GRP’ indicates the location of a cluster of genes encoding ribosomal proteins, almost all of which are transcribed co-directionally with replication. **(B)** Structure of Tus-*ter* (PDB ID: 2I06) (Singleton et al., 2001). **(Bi)** Illustration of the “locked” conformation formed by DNA unwinding at the nonpermissive face. The cytosine base at position 6 of *ter* (C6), which flips into a specific binding site on the nonpermissive face of Tus to form the ‘lock’, is indicated, and important amino acid residues contributing to the locked conformation highlighted. **(Bii)** As above, but a transparent view is shown to allow easier visualisation of the DNA in general and the flipped C6 in particular. **(C)** Sequences of *ter* sites *A–J* from *E. coli* MG1655. The *ter* consensus sequence is shown at the top. Base pairs 1–5, 7, and 19–23 show a higher degree of variability and are shaded in a lighter grey. The highly conserved *ter* core (6, 8–18) is highlighted by a darker grey. Tus binding (Toft et al., 2021), *in vivo* fork blocking observed by 2D gel electrophoresis (Duggin and Bell, 2009) and analysis whether a *ter* site is part of an open reading frame (Duggin and Bell, 2009; Goodall et al., 2021) are indicated (see text for further details).

In all bacteria, replication initiation is tightly regulated (Jameson and Wilkinson, 2017; Katayama et al., 2017; Hansen and Atlung, 2018) and restricted to a single origin (*oriC*) per chromosome (Gao and Zhang, 2008; Gao, 2015). Two replisomes are recruited at *oriC* by the main initiator protein, DnaA, forming two replication fork complexes that bidirectionally duplicate the genome. Bidirectional synthesis continues until the replication fork complexes fuse in an area opposite *oriC* where replication terminates (Masters and Broda, 1971; Prescott and Kuempel, 1972; Wake, 1972, 1973; Gyurasits and Wake, 1973). For bacteria with linear chromosomes replication ceases when fork complexes reach the chromosome ends (Cui et al., 2007; Rudolph et al., 2013). In both cases, the chromosome is divided into two replichores, each replicated with a defined directionality (Figure 1A).

The fact that replication is restricted to two replication fork complexes dictates that, for bacteria with a circular chromosome, each round of chromosome duplication is concluded by exactly one termination event in an area opposite *oriC*. For termination two main mechanisms were initially considered in the literature: the free fusion of moving forks, or site-specific termination. Forks may proceed around the chromosome freely and fuse wherever they happen to meet. Alternatively, there might be distinct locations where fork progression is blocked, forcing fork fusion to take place in a distinct location (Masters and Broda, 1971; Bird et al., 1972; Hendrickson and Lawrence, 2007; Kono et al., 2012).

Replication fork complexes will encounter a wide variety of potential blocks on their way to the terminus area, such as stable protein-DNA complexes, G4 quadruplex DNA or DNA lesions (Lang and Merrikh, 2018; Marians, 2018; Bianco, 2020; Linke et al., 2021). Many of these obstacles are stochastic in nature and can occur at different chromosomal locations for each round of genome duplication. However, there are also mechanisms that have specifically evolved to arrest replication complexes (Hyrien, 2000; Rothstein et al., 2000; Labib and Hodgson, 2007; Hizume and Araki, 2019). The first examples of distinct blocking points for replication complexes were identified in plasmid systems. Replication of the conjugative R plasmid R6K was reported to be either unidirectional or bidirectional (de Graaff et al., 1978), based on a complex mechanism of replication initiation (Abhyankar et al., 2003, 2004; Rakowski and Filutowicz, 2013), but progression of both the clockwise and counter-clockwise forks is blocked by a specific terminus (Crosa et al., 1976). In the R1 plasmid, a distinct termination system is in use to specifically enforce unidirectional replication. Between the minimal origin, *oriR*, and the start site for leading strand replication, two *ter* sites are located, *terR_L_* and *terR_R_* (Hill et al., 1988b). *terR_L_* blocks the counter-clockwise fork almost immediately. Thus, the plasmid is replicated unidirectionally *via* the clockwise fork until it is blocked at *terR_R_* (Krabbe et al., 1997).

The first chromosomal fork block was identified in *E. coli* (Kuempel et al., 1977; Louarn et al., 1977), and shortly afterwards in *B. subtilis* (Weiss et al., 1981). In *E. coli* the integration of an ectopic replication origin into the chromosome was used to show that progression of replication forks was blocked in the termination area (Kuempel et al., 1977; Louarn et al., 1977, 1979). By using a temperature-sensitive version of the main replication initiator protein DnaA to inactivate *oriC*, and inserting an ectopic P2 prophage origin to drive replication initiation at a site 1 Mbp away from *oriC*, the authors showed that termination still occurred diametrically opposite *oriC*, revealing that progression of replication must be impeded in an area opposite *oriC* (Kuempel et al., 1977; Louarn et al., 1977, 1979). Prompted by these results, the use of sophisticated marker frequency analysis revealed specific termination sites, initially called T1 and T2 and later changed to *ter*, in line with plasmid-based systems such as R6K and R1, with the Louarn group identifying the innermost *ter* sites *terA* and *terC*, and the Kuempel lab identifying *terA* and *terB* (Figure 1A; de Massy et al., 1987; Hill et al., 1987, 1988a; Hill and Marians, 1990). In addition, the Kuempel lab identified a region close to *terB* that was needed for *ter* sites to act as a block, and they named the terminus function *termination utilization substance* (*tus*) (Hill et al., 1988a). Further analysis revealed the sequence of the *tus* gene (Hill et al., 1989), and *in vitro* work by Thomas Hill and Ken Marians showed how the binding of Tus protein to a *ter* sequence can arrest progression of DNA replication (Hill and Marians, 1990; Figure 1B).

By developing *ter* consensus sequences, the use of radioactively labeled probes allowed identification of additional *ter* sites *D*, *E*, and *F* (François et al., 1989; Hidaka et al., 1991; Sharma and Hill, 1992). Once the whole genome sequence of *E. coli* was available (Blattner et al., 1997), bioinformatics analysis resulted in the identification of *terG–terJ* (Neidhardt et al., 1996). Thus, the *E. coli* chromosome is divided into two replichores, one replicated clockwise and the other counter-clockwise, with 5 *ter* sites being oriented to block progression of the clockwise replication fork, while the other 5 *ter* sites block the counter-clockwise moving replication fork complex (Figure 1A). Finally, bioinformatics analysis also allowed the identification of the putative and weak *ter* sites *K, L, Y*, and *Z* (Duggin and Bell, 2009).

The specific termination site identified in the Gram-positive *Bacillus subtilis* (Weiss et al., 1981) was later characterized to consist of a cluster of *ter* sites blocking progression of the clockwise replication fork complex, while a second cluster blocked progression of the counter-clockwise replication fork complex (Wake, 1997; Neylon et al., 2005; Rudolph et al., 2019). While acting in a similar fashion, the *ter* sequences show very little similarity to the *E. coli* sequences. Moreover, the *B. subtilis* terminator protein, RTP, has little structural similarity to Tus. Importantly, these differences indicate that the two termination systems developed *via* convergent evolution (Franks et al., 1995; Neylon et al., 2005).

In this review we will discuss how the architecture of bacterial chromosomes limits pathological consequences that can arise when replication forks fuse and explore why a fork trap in the termination area might provide an evolutionary benefit by limiting the local and global impacts caused by fork fusions. Based on the pathological consequences that can arise as a result of fork fusions, we suggest that the strict use of only a single origin per chromosome observed in all bacteria helps to reduce the number of termination events to exactly one, thereby limiting pathologies triggered by fork fusions.

### Mechanism of fork arrest by Tus-*ter* in *Escherichia coli*

How can a single Tus-*ter* complex be such an efficient block to the progression of replication fork complexes? Functional *ter* sites bound by the monomeric terminator protein Tus block replication in a polar manner. Key to this polar activity is that Tus binding is asymmetric, a feature that has been extensively studied (Neylon et al., 2005; Elshenawy et al., 2015; Pandey et al., 2015; Berghuis et al., 2018). Arrest of an approaching replication fork complex is triggered when the double-stranded DNA immediately adjacent to one face of Tus, called the non-permissive face, is unwound as the fork approaches (Mulcair et al., 2006). The structural change in the opened double helix induces specific contacts between the DNA and Tus that result in the formation of ‘locked complex’, which will block progression of the oncoming replisome with surprising efficiency, a mechanism referred to as a ‘mousetrap’ (Mulcair et al., 2006). Specifically, a base on the leading strand template (C in position 6) flips into a binding site on Tus, guided by nearby residues (Figure 1B and Table 1). This mechanism of ‘clicking into place’ essentially blocks any movement along the DNA by a ratchet-type mechanism, and also slows the dissociation of Tus (Mulcair et al., 2006; Berghuis et al., 2015; Elshenawy et al., 2015; Pandey et al., 2015). This ‘locked’ Tus-*ter* complex can be generated by unwinding of the double-stranded DNA alone; DnaB helicase is not essential for its formation (Bastia et al., 2008; Berghuis et al., 2015; Elshenawy et al., 2015; Toft et al., 2022). Some stabilization by specific protein–protein interaction between Tus and DnaB has been reported (Mulugu et al., 2001), but to what extent they contribute towards the efficient arrest is not clear. Multiple labs have highlighted key residues which have major roles in the formation of a successful Tus-*ter* lock (Table 1). Mutants in these residues alter the C6 binding, ssDNA guiding, and slowing the momentum of the approaching replisome.

**Table 1:** Key amino acid residues of *E. coli* Tus protein and their role in blocking progression of replication fork complexes.

Residue	Importance	Reference
E47	Stabilize ‘mousetrap’	Mulugu et al. (2001) and Toft et al. (2021)
E49	Slow approaching fork *via* helicase interaction	Berghuis et al. (2015)
F140	Holding C6 in place	Mulcair et al. (2006)
H144	Holding C6 in place	Mulcair et al. (2006)
G149	Holding C6 in place	Mulcair et al. (2006)
R198	Slow approaching fork *via* helicase interaction	Elshenawy et al. (2015)

If individual Tus-*ter* complexes block replication progression so effectively, why are so many *ter* sites found in the *E. coli* chromosome? It has become increasingly clear that not all sequences identified as *ter* sites block forks. Also, even a *ter* site with a strong blocking activity is not an absolute block. In a study using 2-dimensional neutral-neutral gel electrophoresis analysis, Duggin and Bell were able to show that in wild type cells the vast majority of blocked forks occur at the innermost *ter* sites of the fork trap system. Highest levels were detected at *terC* and *terB*, followed by *terA.* In this study rather mild levels of signal were found for *terD* and *terG* (Figure 1C), and the lowest levels were identified for *terH* and *I*, while *ter* sites *E*, *F* and *J* showed no signal in wild type cells (Duggin and Bell, 2009). In a recent study by the Schaeffer lab, ChiP-Seq was used to identify which *ter* sites are actually occupied by Tus protein. The authors identified *ter* sites *A–E* and *G* (Figure 1C), whereas no Tus occupancy was observed at any of the other *ter* sites (Toft et al., 2021).

**Figure 2:**
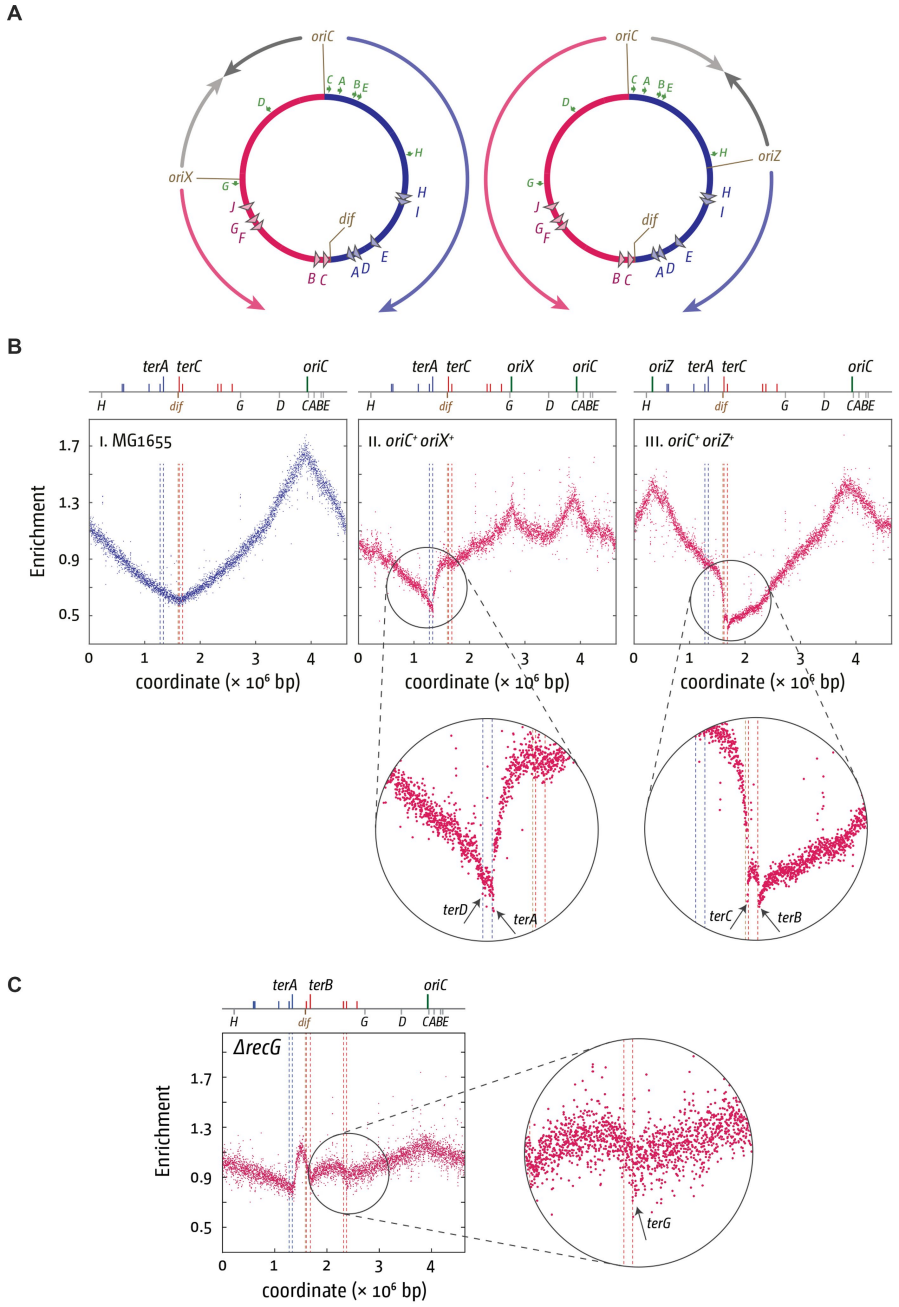
Altered replichore structure in *E. coli* cells with additional ectopic replication origins. **(A)** Schematic representation of *E. coli* chromosomes with additional ectopic replication origins *oriX* (left) and *oriZ* (right), respectively. Positions of *oriC*, *ter* sites as well as the *dif* site and *rrn* operons *A–E, G*, and *H* are shown. **(Bi–iii)** Marker frequency analysis (MFA) in MG1655, *oriC^+^ oriX^+^* and *oriC^+^ oriZ^+^* cells. The number of reads (normalized against reads for a stationary phase wild type control) is plotted against the chromosomal location. A schematic representation of the *E. coli* chromosome showing positions of *oriC, oriX* and *oriZ* (green lines) and *ter* sites (all above) as well as *dif* and *rrn* operons *A–E, G*, and *H* (all below) is shown above the plotted data. The MFA raw data were taken from Ivanova et al. (2015) and Dimude et al. (2018b) and re-plotted to allow axis scale changes, if necessary. The areas where forks are blocked by Tus-*ter* complexes are highlighted by circles and details magnified below to highlight the fork blocking ability of *ter* sites *A, B, C*, and *D*. **(C)** Over-replication in the termination area of *ΔrecG* cells growing in M9 minimal salts with glucose. The area where forks escaping the innermost *ter* sites are blocked by Tus-*ter* complexes are highlighted by a circle, and details magnified to the side to highlight the fork blocking ability of *ter* site *G*. The MFA raw data were taken from Midgley-Smith et al. (2018) and re-plotted.

These observations fit well with our own data. The replichore arrangement can be significantly skewed by introducing additional ectopic copies of the *oriC* sequence into the chromosome. In a strain with two active replication origins (Wang et al., 2011; Ivanova et al., 2015; Dimude et al., 2018b), one fork reaches Tus-*ter* complexes much earlier than the other. Depending on whether the ectopic origin is located in the left-hand or right-hand replichore, forks get arrested at *terC/B* or *terA/D* (Figure 2A), which can be visualized by using high-resolution marker frequency analysis (MFA) from whole genome sequencing (WGS) (Ivanova et al., 2015; Dimude et al., 2018b; Syeda et al., 2020). These replication profiles, generated by WGS, reveal initiation sites of synthesis, such as *oriC*, as distinct peaks, whereas termination sites form a v-shaped valley, with a replication gradient between initiation and termination sites (Skovgaard et al., 2011; Figure 2Bi). Our MFA analyses confirm that the four innermost *ter* sites *A–D* form a strong block. All four are low points in the replication profile (Figures 2Bii,iii), in line with the idea that individual Tus-*ter* complexes can be overcome occasionally, but the strong ‘step’ in the replication profile confirms the considerable block that is formed, especially by the innermost *ter* sites *A* and *C/B*.

Additional information comes from the replication profiles of strains that show over-replication of the termination area. Over-replication of the chromosome can be triggered by the absence of certain proteins, such as Tus, RecG or 3′ exonucleases, resulting in a peak of synthesis between *ter* sites *A* and *B/C* (Markovitz, 2005; Duggin et al., 2008; Rudolph et al., 2009, 2013; Wendel et al., 2014; Midgley-Smith et al., 2019). Synthesis can proceed beyond *ter* sites *C* and *B* under certain conditions in cells lacking RecG helicase (Figure 2C; Midgley-Smith et al., 2018). The precise molecular mechanism for this regular progression of synthesis beyond *ter* sites *C* and *B* is not clear. However, the ChIP-Seq data from the Schaeffer lab would predict that escape synthesis should proceed past *terF* before getting blocked by *terG* (Toft et al., 2021). This is indeed precisely what we observed, supporting the idea that *terF* is not blocking the progression of forks in normal cells, whereas *terG* can block ongoing synthesis in wild type cells (Figure 2C). Thus, while the majority of the work of blocking forks is done by three of the innermost *ter* sites *A* and *B/C*, with *terD* clearly being active (Figure 2B) but contributing relatively little, as observed before (Duggin and Bell, 2009), some progressing synthesis can also be blocked by more distal *ter* sites, such as *terG*.

Given that the innermost Tus-*ter* complexes in particular very efficiently block progression of ongoing synthesis, the fusion of replication fork complexes will occur almost exclusively within the innermost termination area. However, this does not answer the question of whether Tus-*ter* complexes are a regular component of termination, or if they are only involved when progression of the two replication fork complexes becomes asynchronous, for example if one of the two complexes is arrested by a DNA lesion, a secondary structure or a protein-DNA complex for any length of time.

Early DNA labeling experiments demonstrated that fork fusion events typically occur close to the numerical midpoint of the chromosome and away from Tus-*ter* complexes (Bouché et al., 1982). This finding is supported by our own MFA-based replication profiles. MFA-based replication profiles use 1 kb windows per individual data point, which means resolution is relatively coarse (Skovgaard et al., 2011; Rudolph et al., 2013). However, within the limits of this resolution, our data demonstrate that the fork fusion point, as determined by LOESS regression, is in precisely the same location in the presence and absence of Tus protein (Rudolph et al., 2013; Dimude et al., 2016). The only change observed is a mild distortion of the marker frequencies in the vicinity of the fusion point (see Dimude et al. (2016) and Goodall et al. (2021) for a direct comparison of the replication profiles in the presence and absence of Tus terminator proteins). In contrast, in cells in which the replichore arrangement is artificially skewed by an additional ectopic origin, the fork fusion point is significantly shifted if the *tus* gene is deleted, as expected (Ivanova et al., 2015; Dimude et al., 2018b; Syeda et al., 2020). These findings suggest that a significant proportion of fork fusion reactions take place in a relatively short stretch between the *dif* chromosome dimer resolution site and *terB*, regardless of whether Tus is present or absent (Rudolph et al., 2013; Ivanova et al., 2015; Dimude et al., 2016). However, the fact that the termination area changes shape in the replication profile of cells lacking Tus demonstrates that Tus-*ter* complexes do contribute to termination on a regular basis (Rudolph et al., 2013; Dimude et al., 2015). Indeed, in a modelling study by Kono and colleagues it was demonstrated that a mixture of Tus-*ter* based and free fusion reactions was shown to result in GC-skew profiles that represented the experimental data most closely (Kono et al., 2012). Additionally, the authors did observe significant differences of the GC-skew profiles in bacteria which do not have a Tus or RTP-based fork trap system (Kono et al., 2012). Taken together, the data suggest that replication often terminates *via* the free fusion of forks, but if a fork trap system is present this also contributes to the termination process on a regular basis.

### Fork trap systems in other bacterial species

Galli and co-workers recently analyzed the bacterial species that have a Tus-*ter* fork trap system. They showed that the presence of a replication fork trap in the termination area of the chromosome is a specialized feature of a relatively small group of bacteria (Galli et al., 2019). This suggests that perhaps the majority of bacterial species do not have a fork trap mechanism. Proteins with homology to Tus can be found in most Enterobacteriales, the Pseudoalteromonas and most Aeromonadales (Galli et al., 2019). As already highlighted, a fork trap system can be found in *B. subtilis*, but distribution amongst *Bacillus* species is even more restricted and the dissimilarity to Tus-*ter* strongly suggests that its origin is different and has arisen as a result of convergent evolution, highlighting the importance of a fork trap (Franks et al., 1995; Neylon et al., 2005; Galli et al., 2019). Indeed, the binding mechanisms of the terminator proteins to their *ter* sites are different, with Tus being a polar monomer and the *B. subtilis* terminator protein, RTP, being a dimer that requires a conformation change to arrest forks (Eli Bussiere et al., 1995; Franks et al., 1995; Pai et al., 1996; Griffiths et al., 1998; Wilce et al., 2001). In the *B. subtilis* chromosome, terminator sites are formed *via* two ‘half-sites’, the A and the B site, each bound by an RTP dimer (Lewis et al., 1990). Thus, in total four RTP monomers bind to each *ter* sequence (Lewis et al., 1990). However, binding of an RTP dimer to the A site is much weaker than binding of the second dimer to the B site. Consequently, forks arriving at the B site first will be blocked, while forks arriving at the A site can proceed (Smith and Wake, 1992), which is fundamentally different to the *E. coli* Tus-*ter* system. Interestingly, the transcriptional regulation of the genes encoding for RTP and Tus are achieved through auto-regulation, with a *ter* site being located in the promotor area of the respective genes (Natarajan et al., 1991; Ahn et al., 1993).

**Figure 3:**
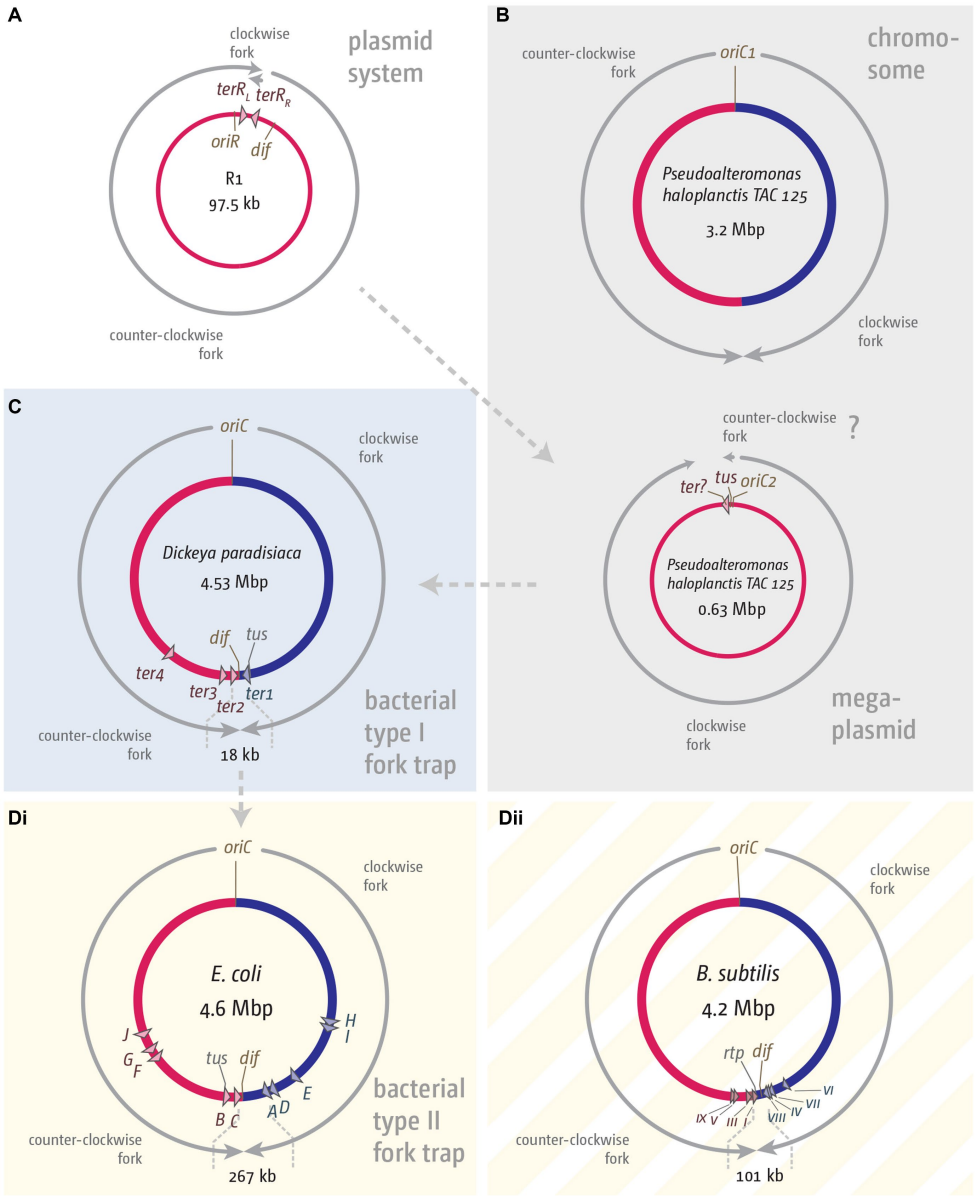
Comparison of fork trap systems in various plasmids and bacterial chromosomes. **(A)** Replication dynamics and fork trap system in the plasmid R1. **(B)** Replication and fork trap features of the primary and secondary chromosome in *Pseudoalteromonas haloplanctis* TAC 125. The secondary chromosome contains a *tus* gene, which is located next to the origin of replication, *oriC2*. The presence of a *ter* site is only implicated and therefore marked with a question mark. **(C)** Type I fork trap system in *Dickeya paradisiaca*, as described in Toft et al. (2022). **(D)** Type II fork trap system in *E. coli* **(Di)** and the fork trap system in *Bacillus subtilis* **(Dii)**. See main text for further details.

The phylogenetic analysis by Galli and co-workers resulted in the interesting hypothesis that the *E. coli* fork trap system might have been domesticated from plasmid-based precursor systems such as in R1 (Figure 3A). The authors highlight that in the Pseudoalteromonadacae, *tus* is always located directly adjacent to the origin of the secondary chromosome, which is a mega plasmid (Galli et al., 2019; Figure 3B). Indeed, the GC skew for the secondary chromosome in *Pseudoalteromonas haloplanktis* fits a unidirectional replication mode (Médigue et al., 2005; Xie et al., 2021). A fork trap in close proximity to the origin, together with the unidirectional mode of replication, is indeed very reminiscent of the system found in the R1-type plasmids (Figure 3A; Krabbe et al., 1997).

If the fork trap system was domesticated in this fashion, it is easy to imagine that the unidirectional mode of replication will become rate-limiting for the size of chromosomes, a problem easily solved by a ‘remodelling’ of the chromosome that resulted in the integration of sequences between the origin and the Tus-*ter* complexes (Figure 3C). A prediction from this hypothesis would be that there might be chromosomes with a single *ter* site limiting progression of one of the replichores, and where the *tus* gene is associated with this innermost *ter* site. In a recent study by Toft and colleagues it was indeed highlighted that two distinct fork trap architectures exist (Toft et al., 2021). The fork trap system in *E. coli* is classed as a type II system, because the *ter* site regulating transcription of the *tus* gene is *terB*, which is a secondary rather than the innermost *ter* site (Toft et al., 2021). Outside of the Enterobacteriaceae, another system exists which the authors classified as a type I system. In organisms such as *Edwardsiella tarda* or *Dickeya paradisiaca* a single *ter* site is found to block progression of one replichore, and also acts as a transcriptional repressor of the *tus* gene (Toft et al., 2021, 2022), exactly as predicted. In organisms such as *Edwardsiella tarda* and *Cedecea neteri* the second replichore is also restricted by only a single *ter* site, whereas in organisms such as *Dickeya paradisiaca* two functional *ter* sites are used (Toft et al., 2021, 2022). The authors speculate that the type I system is the ancestral system, whereas the type II system is more divergent (Toft et al., 2021, 2022), which seems overall to fit well with the notion of a plasmid-derived system (Figure 3; Galli et al., 2019).

However, while the classification into a type I and II system is helpful to some degree, the systems are likely to be more similar than they appear upon casual inspection. There is no doubt that the number of innermost *ter* sites differ across species, together with the association and relative location of the *tus* genes. But the outer *ter* sites *F–J* in *E. coli* are all part of open reading frames, which means that, regardless of whether it ever was a functioning *ter* site or whether the sequence similarity is coincidence, the sequence will be conserved as part of the maintenance of the genes they are in. Both arguments are not mutually exclusive, but the ChIP-Seq data from the Schaeffer lab highlight that the occupancy of *ter* sites *F* and *H–J* is low (Toft et al., 2021). Indeed, when we analyzed the sequence conservation of all *ter* sites across all phylogenetic groups in *E. coli*, we found a revealing trend: for the innermost *ter* sites *A–D*, which clearly have a role in blocking forks (Duggin and Bell, 2009; Toft et al., 2021), strict conservation was observed for all bases that make contact with Tus. However, especially at bases 1–5 and 7 numerous variations were found, which are unlikely to affect Tus binding (Goodall et al., 2021). Interestingly, the same pattern was observed for *terG* (Goodall et al., 2021), the one *ter* site which is not part of the inner fork trap, but for which Tus binding and significant fork blocking activity was demonstrated (Duggin and Bell, 2009; Midgley-Smith et al., 2018; Toft et al., 2021). For *ter* sites *E*, *F* and *H–J* far fewer sequence variations were observed, a trend that extends beyond the 23 bp *ter* sequence (Goodall et al., 2021), in line with the idea that the sequence conservation might be driven to a large extent by the ORF they are in Table 2.

**Table 2:** Analysis of various parameters of all predicted *ter* sites in the *E. coli* chromosome.

Site	Chromosomal location [Mbp]	Within ORF	Sequence conservation	Fork block	References
***terA***	1.34	mixed	1 SNP/23 bp	**Yes**	Duggin and Bell (2009), Ivanova et al. (2015), Dimude et al. (2018b), and Toft et al. (2021)
***terB***	1.68	No	1–2 SNPs/23 bp	**Yes**	
***terC***	1.61	No	1–2 SNPs/23 bp	**Yes**	
***terD***	1.28	No	1–2 SNPs/23 bp	**Yes**	
*terE*	1.08	No	0 SNP/23 bp	Not observed	Duggin and Bell (2009)
*terF*	2.32	Yes	1 SNP/23 bp	No	Duggin and Bell (2009), Midgley-Smith et al. (2018), and Toft et al. (2021)
*terG*	2.38	Yes	1–2 SNPs/23 bp	**Yes**	Duggin and Bell (2009), Midgley-Smith et al. (2018), and Toft et al. (2021)
*terH*	0.60	Yes	0 SNP/23 bp	No	Duggin and Bell (2009) and Toft et al. (2021)
*terI*	0.625	Yes	1 SNP/23 bp	No	Duggin and Bell (2009) and Toft et al. (2021)
*terJ*	2.58	Yes	1 SNP/23 bp	No	Duggin and Bell (2009) and Toft et al. (2021)
*terK*	2.28	Yes	nd^a^	No	Duggin and Bell (2009)
*terL*	2.88	Yes	nd^a^	No	Duggin and Bell (2009)
*terY*	2.87	No	5 SNPs/23 bp	No	Duggin and Bell (2009)
*terZ*	3.44	Yes	nd^a^	No	Duggin and Bell (2009)

^a^We did not systematically determine the sequence variations across the *E. coli* phylogroups for ter sites *K*, *L*, and *Z*.

In this analysis *terE* stands out, because it is not part of an ORF (Table 2), but is still highly conserved (Goodall et al., 2021). *terE* is located within the *efeUOB* operon, which, in enterohaemorrhagic *E. coli* O157:H7, encodes an iron transport system (Cao et al., 2007). *terE* is located between *efeU*, a pseudo-gene in *E. coli* K12 (Grosse et al., 2006), and *efeO*. In our sequence analysis across all *E. coli* phylogroups we did not find any variations of *terE*, nor of the wider region around the actual *terE* sequence (Goodall et al., 2021). This conservation makes it tempting to speculate that it is located in a sequence environment with an important regulatory function, even though we do not know what this function is. In contrast, when we analyzed *terY*, a pseudo *ter* site that neither acts as a part of the fork trap nor is it located within an ORF (Table 2), we observed significant sequence variations across the phylogenetic groups, as well as changes in its relative location, in line with the idea that this sequence snippet is subjected to unselective evolutionary drift (Goodall et al., 2021).

Taken together, the data suggest that we can simplify the fork trap system in *E. coli* and related bacteria by focusing on the innermost *ter* sites *A–D* as those being active in termination. The main difference between the type I and type II systems remains whether the border of the fork trap is defined by a single *ter* site associated with the *tus* gene (type I), or whether a cluster of *ter* sites is used, with *tus* associated with one of the secondary *ter* sites (type II) (Toft et al., 2022). All of the other *ter* sites in *E. coli* are likely to be still present because of selection pressures unrelated to termination issues.

### Fork trap systems can pose problems for DNA replication

The defined architecture of the bacterial chromosome, where a single origin results in each replichore being replicated by a single replication fork complex, dictates that any restriction to fork movement will become problematic if a fork is held up unexpectedly, for example at a small DNA lesion or a protein-DNA complex (Lang and Merrikh, 2018; Marians, 2018; Bianco, 2020; Linke et al., 2021). Any stalled fork has to be reactivated to complete chromosome replication, as the only other fork available is blocked by Tus-*ter* complexes, a clear disparity to the concept of dormant origins in eukaryotic cells (Blow et al., 2011; Shima and Pederson, 2017). If fork reactivation cannot be achieved and the other fork is arrested by the multiple Tus-*ter* complexes in its way, the chromosome will remain only partially replicated, with potentially lethal consequences for the cell.

The difficulties caused by this scenario can be shown experimentally in cells in which the replichore arrangement is distorted. This was done in *B. subtilis* in which a *ter* site was integrated in blocking orientation into an ectopic location of the chromosome, on the edge of the replication fork trap system. Forks were blocked for a significant period of time, causing significant morphological changes of the affected cells (Franks and Wake, 1996). Indeed, similar experiments were done in *E. coli* by the Michel lab, showing that arrested forks only can be reactivated if a second round of replication reaches the arrested primary fork, resulting in the generation of linear DNA molecules that engage in homologous recombination and allow reactivation of forks. If recombination is inactivated, the presence of an ectopic *ter* site proved lethal (Bidnenko et al., 2002, 2006).

In our own work we used strains where, instead of using ectopic *ter* sequences, the origin was moved 1 Mbp into an ectopic position. In this scenario one of the two forks initiated will duplicate ¼ of the chromosome before being arrested at Tus-*ter* complexes, while the other fork has to duplicate the remaining ¾ of the chromosome. In addition, this fork has to replicate ¼ of the chromosome in an orientation opposite to normal. The doubling time of such a construct was approximately 40 min, twice that of the 20 min normally observed for wild type cells (Ivanova et al., 2015). Inactivation of the replication fork trap by the deletion of *tus* reduced the doubling time to ~30 min (Ivanova et al., 2015). Together, the findings in these experimental systems strongly highlight the impact the replication fork trap has to impede the movement of replisomes (Franks and Wake, 1996; Bidnenko et al., 2002; Ivanova et al., 2015).

**Figure 4:**
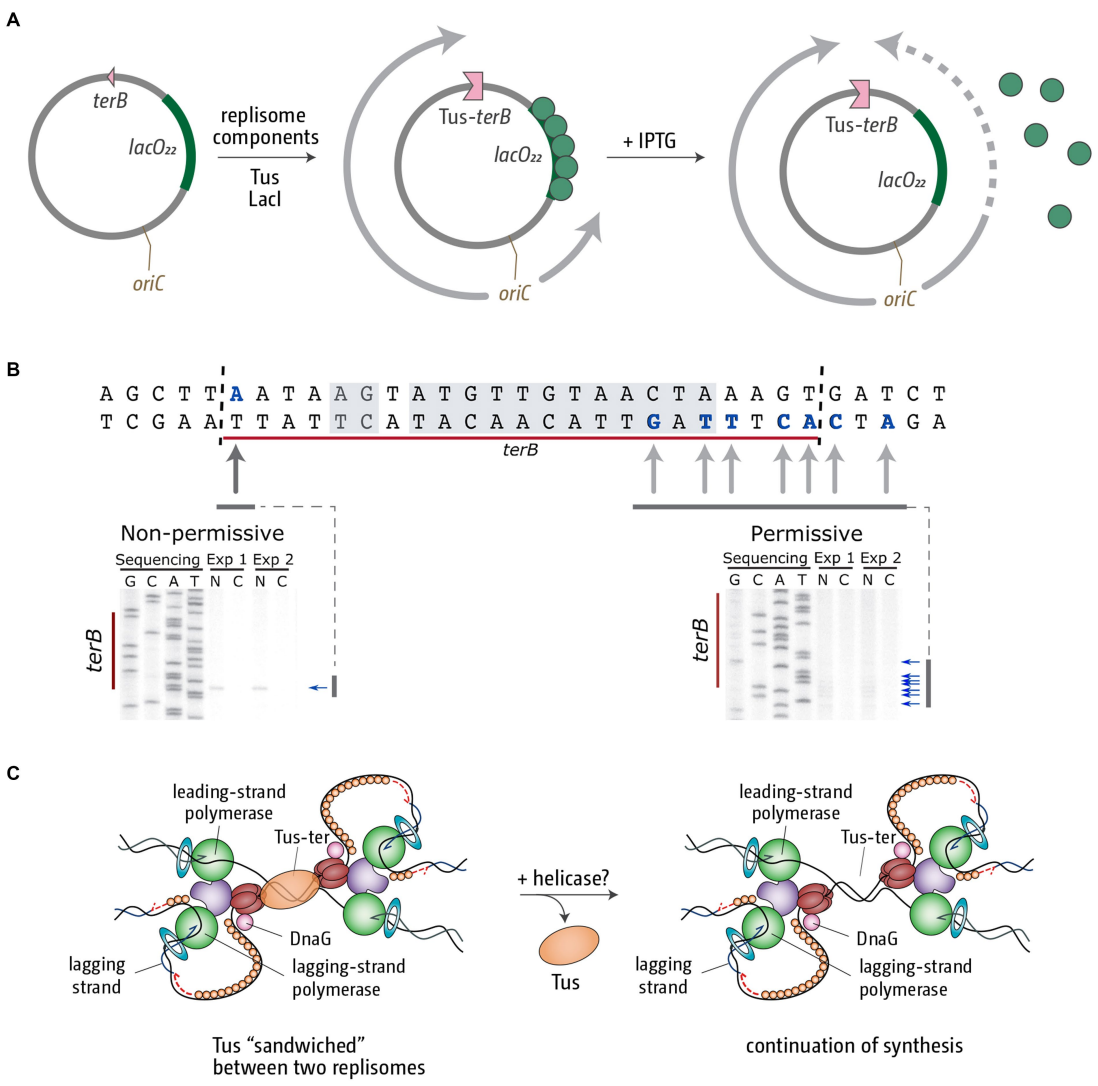
Replication fork fusion can be controlled *in vitro* to occur at Tus-*terB*. **(A)** pKJ1 replication assay template, indicating the location of replication initiation at *oriC*, the *terB* site and the *lacO22* array. Replisome movement is shown pre (middle) and post (right) LacI-*lacO* block removal. **(B)** *terB* sequence indicating leading strand stop locations (bold and blue) and the Tus-binding site (shaded area). Mapping analysis of the leading strand products approaching Tus-*terB* in the non-permissive and permissive direction is shown below. Nicked products (stop sites) are indicated by arrows. Mapping data were reproduced from Jameson et al. (2021). **(C)** Schematic representation of the termination intermediates generated by a fork fusion event taking place at Tus-*terB*. We believe the most likely candidate for the removal of Tus from this super-complex *in vivo* is an as yet unidentified helicase.

However, even if one fork is blocked at an obstacle only for a limited period of time, the replication fork trap still poses a threat to the completion of DNA synthesis. At fork speeds of 700–1,000 nt/s (Baker and Bell, 1998; Pham et al., 2013) even a short delay of one of the forks will cause the second to get arrested at a Tus-*ter* complex. This means that, upon reactivation, the temporarily arrested fork will eventually reach a replication fork complex that is stably arrested at a Tus-*ter* complex. While the arrest of a single replisome by Tus-*ter* complexes has received considerable attention, and a substantial number of studies have looked at the effects of Tus-*ter* on the progression of reconstituted replication fork complexes *in vitro* (Khatri et al., 1989; Lee et al., 1989; Hill and Marians, 1990; MacAllister et al., 1990; Gottlieb et al., 1992; Hiasa and Marians, 1994; Neylon et al., 2000), the fusion reaction of a freely moving fork with another stably arrested at a Tus-*ter* complex has not really been studied, even though this is likely to be physiologically very relevant. We were recently able to reconstitute this specific reaction *in vitro*. We used a plasmid template with an *oriC* sequence which, upon adding all the purified *E. coli* replisome components, together with the DnaA and DnaC initiator proteins, allows the formation of fully functional replication fork complexes (Jameson et al., 2021). The plasmid substrate contains *terB*, which, upon addition of Tus, will block progression of one of the two established forks (Figure 4A). The second fork moving in the opposite direction can be blocked at an array of 22 copies of the *lacO* sequence if the *lac* repressor protein LacI is added. The block is temporary, because addition of IPTG allows release of the block and continuation of synthesis until the second fork reaches the Tus-*ter* complex from the permissive side (Figure 4A). This precisely mimics the scenario of a moving fork running into another stably arrested at a Tus-*ter* complex (Jameson et al., 2021).

Termination mapping analysis revealed that in this scenario the template also remains under-replicated. We observed a gap of between 15 and 24 bp, which matches the footprint of Tus (Figure 4B; Jameson et al., 2021). Thus, it is very tempting to speculate that, as a result of this particular type of fusion reaction, Tus remains stably bound, sandwiched between two replication fork complexes (Figure 4C; Jameson et al., 2021). We currently do not know how this complex is resolved *in vivo*. It seems likely that a helicase needs to be involved to remove the bound Tus protein and facilitate progression of at least one of the two forks to facilitate completion of DNA synthesis (Figure 4C). However, we do not currently know which helicase might be involved in this reaction (Jameson et al., 2021).

Nevertheless, both scenarios highlight that, within the defined architecture of the *E. coli* chromosome, a fork trap poses a risk that can prove fatal. We therefore wanted to investigate whether we could find a fork trap system that was inactivated by point mutations in specific *E. coli* strains. *E. coli* can be found in the gastrointestinal tract of a wide variety of warm-blooded animals (Stoppe et al., 2017). These populations are shaped by factors specific to the host as well as the environment, resulting in a high degree of diversity. This diversity is reflected in a core genome that contains approximately 2,200 genes, while the pan-genome contains more than 13,000 genes (Rasko et al., 2008). However, despite this variability, our sequence analysis did not reveal even a single example of a phylogenetic group where the replication fork trap appears to be non-functional (Goodall et al., 2021). Thus, while a fork trap clearly is not essential, and a wide variety of bacterial species appear not to utilize a fork trap (Galli et al., 2019), it appears that, once acquired, such a fork trap system is strictly maintained (Neylon et al., 2005; Goodall et al., 2021; Toft et al., 2022). Together with the fork trap system in *B. subtilis*, which has a similar functionality but has likely arisen by convergent evolution (Franks et al., 1995; Neylon et al., 2005), these findings suggest that a fork trap system is involved in mitigating a problem important enough to selectively maintain the fork trap system.

### Are replication-transcription conflicts responsible for the evolution of fork traps?

One proposed role for the fork trap is maintaining the co-directionality of transcription and replication (Brewer, 1988). Head-on collisions of replication and transcription complexes pose a challenge to cells (Rudolph et al., 2007; Kim and Jinks-Robertson, 2012; McGlynn et al., 2012; Merrikh et al., 2012; Lang and Merrikh, 2018), a challenge that becomes especially significant if collisions occur at highly-transcribed genes such as the operons for ribosomal RNA (Wang et al., 2007; Boubakri et al., 2010; Srivatsan et al., 2010; De Septenville et al., 2012; Ivanova et al., 2015; Dimude et al., 2018a; Hawkins et al., 2019). Especially highly transcribed genes, such as *rrn* operons and genes encoding ribosomal proteins, are transcribed co-directionally with the direction of replication (Brewer, 1988; McLean et al., 1998). If a fork would proceed beyond the termination area, perhaps because the other is held up at an obstacle, the proceeding fork will now encounter these genomic areas in the dangerous head-on orientation, which is normally prevented by a fork trap.

Is this likely to be the driver behind the strict maintenance of a fork trap area? In *E. coli* all *rrn* operons and the majority of genes encoding ribosomal proteins are located in the origin-proximal half of the chromosome (McLean et al., 1998; Dimude et al., 2016). Forks proceeding from the termination area in the wrong orientation would have to duplicate 1 Mbp and more before they would even reach these highly transcribed areas. At least under laboratory conditions, and within the resolution limit of the MFA used, the fork fusion point is surprisingly consistent (Rudolph et al., 2013; Dimude et al., 2016; Goodall et al., 2021), which is perfectly in line with the relatively small percentages of stalled forks detected by 2D gel electrophoresis (Duggin and Bell, 2009). Together, this suggests that long delays of a single fork are a rare event.

For the rest of the *E. coli* chromosome co-directionality is approximately 55% (McLean et al., 1998). However, this does not necessarily mean that directionality is random. Data from Merrikh and colleagues have shown that head-on transcription-replication conflicts increase the incidence of mutagenesis that can result in adaptive mutations, which actually increase cell survival (Merrikh et al., 2012, 2016; Merrikh, 2017; Merrikh and Merrikh, 2018). This arises from the fact that many stress-induced genes are encoded on the lagging strand, thereby triggering head-on collisions with transcription machinery and, as a result, location-specific mutagenesis (Schroeder et al., 2016, 2020). Thus, while co-directionality reduces conflicts and for this reason is favored for highly transcribed genes, head-on collisions might be advantageous during times of stress, as they are useful for adaptive mutagenesis (Lang and Merrikh, 2018; Schroeder et al., 2020). Still, these effects are likely to be more subtle than the consequences of head-on collisions at highly-transcribed locations.

To gain at least some insight into whether forks escaping the termination area are causing any systematic problems before reaching the highly-transcribed *rrn* operons we used cells which contain ectopic replication origins in addition to *oriC*, as described above, and in which the fork trap is inactivated by the deletion of the *tus* gene. In Δ*tus* cells carrying an ectopic replication origin called *oriZ* roughly half way into the clockwise replichore, the fork fusion point is shifted significantly into the counter-clockwise replichore, with one fork escaping the termination area and duplicating roughly 500 kb before fusing with the opposing fork (Ivanova et al., 2015). The mirrored situation takes place in Δ*tus* cells carrying an ectopic replication origin called *oriX* roughly half way into the counter-clockwise replichore (Dimude et al., 2018b). Our marker frequency analysis did not reveal any systematic delays of the forks escaping the termination area, very much in contrast to the situation observed at *rrn* operons (Ivanova et al., 2015; Dimude et al., 2018a,b; Hawkins et al., 2019). Thus, we currently do not have any data supporting the idea that avoiding the negative consequences of head-on replication-transcription clashes in the origins-distal half of the chromosome is contributing in a major way to the evolutionary maintenance of the replication fork trap, even though control of directionality imposed by the fork trap almost certainly will be an added bonus. It would be interesting to analyze mutation rates and spectrums in the area replicated opposite to normal to also investigate the potentially beneficial effects of replication-transcription conflicts in this area of the chromosome.

### Making sense of the replication fork trap

#### A role in preventing over-replication

If we assume that the fork trap system was derived from an ancestral system that came from plasmids, it is important to consider what is known about the physiological role of such plasmid-born fork trap systems. As described before, for R1 plasmid replication the fork trap mechanism enforces unidirectional DNA replication. Two *ter* sites, *terR_L_* and *terR_R_*, are located between the minimal origin sequence and the start site for leading strand synthesis (Hill et al., 1988b). DNA replication proceeding counter-clockwise is almost immediately blocked by *terR_L_*, whereas the clockwise fork duplicates most of the plasmid DNA until it is blocked at *terR_R_* (Krabbe et al., 1997). Interestingly, for R1 replication the absence of Tus protein was shown to result in the accumulation of complex branched DNA structures, plasmid multimers and rolling circle replication intermediates, which interfered with successful plasmid segregation (Krabbe et al., 1997). These findings strongly suggest that the replication fork complex, upon encountering the already replicated part of the plasmid, continues to unwind the already generated nascent section if it is not arrested by a Tus-*ter* complex. The resulting intermediates then serve as substrates for the continuation of replication in various ways (Krabbe et al., 1997). This idea is supported by biochemical studies showing that Tus-*ter* complexes forming a fork trap can prevent over-replication of a circular plasmid substrate, both by a traditional *in vitro* study (Hiasa and Marians, 1994) and the recently developed elegant “replication chain reaction” (Hasebe et al., 2018), lending support to a function of the bacterial replication fork trap in preventing over-replication and, as a result, segregation difficulties, which would pose a strong selection pressure.

Is there any indication that the fork trap might have a similar function for the *E. coli* chromosome? It was indeed reported that the deletion of *tus* resulted in low levels of chromosomal over-replication, an effect significantly exacerbated by specific point mutants in the *polA* gene, which encodes for DNA polymerase I (Markovitz, 2005), a polymerase involved in maintenance and repair processes, such as Okazaki fragment maturation and various DNA repair pathways (Kurth and O’Donnell, 2009). We were able to confirm over-replication of the termination area in a strain with a *polA2* point mutation (Midgley-Smith et al., 2018). These data are in line with the idea that the fork trap, together with specific proteins such as polymerase I, is involved in bringing DNA replication to a successful conclusion (Markovitz, 2005; Duggin et al., 2008; Midgley-Smith et al., 2018; Rudolph et al., 2019). Similarly, absence of the terminator protein RTP in *B. subtilis* results in an increased formation of chromosomal dimers, which could arise from a related mechanism (Lemon et al., 2001).

**Figure 5:**
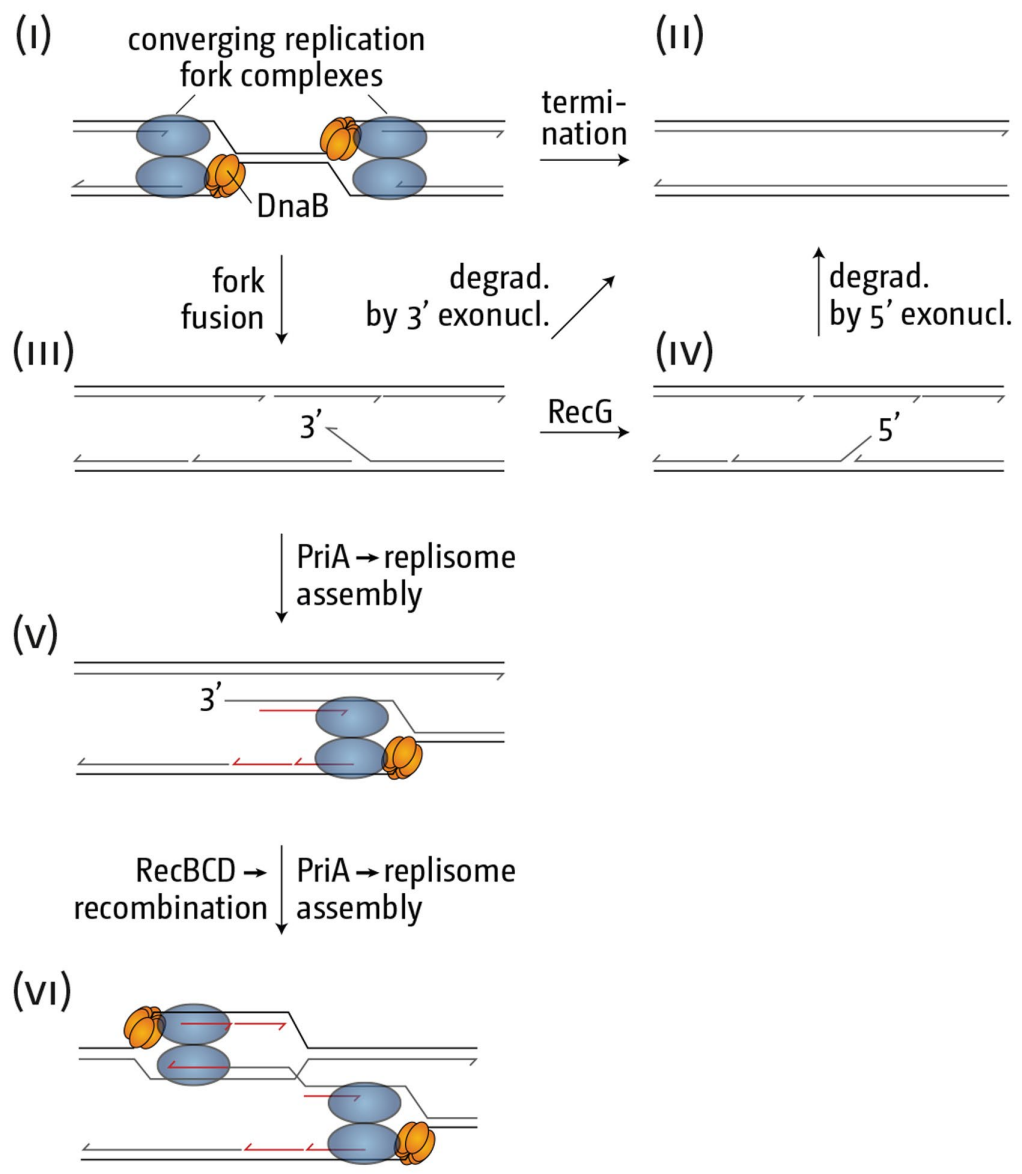
Schematic illustrating how replication fork fusions might trigger over-replication in the termination area. **(i)** Two merging replication fork complexes. **(ii)** Successful termination event where the two replisomes are disassembled, synthesis of all strands is completed and all nascent strands are successfully sealed using DNA ligase. **(iii)** As part of the fork fusion reaction the helicase of one replication fork complex might displace the leading strand polymerase of the opposing fork, resulting in the formation of a 3’ flap structure, which might be degraded by proteins such as 3’ exonucleases, resulting in successful termination. Note that the formation of a 3’ flap can occur at both forks. However, for simplicity the schematic shows only one such reaction. **(iv)** A 3’ flap is one of the best substrates for RecG helicase, which would convert it into a 5’ flap. Upon degradation by 5’ exonucleases successful termination can be achieved. **(v)** If 3’ flaps persist they are also a substrate for the restart protein PriA, which will establish a replisome, thereby not only over-replicating an already fully replicated area of the genome, but also generating a double-stranded DNA end. **(vi)** The dsDNA end can engage in homologous recombination, resulting in the formation of a displacement or D-loops, which PriA will use to set up yet another replication fork complex, thereby exacerbating the problem of over-replication.

What might be the molecular structures that arise as part of termination? Systematic analysis of a number of mutants that show over-replication of the termination area have given important insights. Significant levels of over-replication of the termination area were observed in cells lacking RecG helicase (Rudolph et al., 2009, 2013; Wendel et al., 2014; Dimude et al., 2015), an effect that is completely suppressed by point mutations inactivating the helicase activity of the main replication restart protein PriA (Rudolph et al., 2013; Dimude et al., 2015). Even more specifically, the over-replication is suppressed in a PriA point mutation called *srgA1*, encoding PriA[L557P], a PriA protein with a very specific change of the substrate activity. It unwinds a replication fork with both a leading and a lagging strand at the branch point as efficiently as wild type PriA, but it has lost the ability to unwind a fork in which the leading strand is missing (Gregg et al., 2002), which is, effectively, a 3′ flap structure. This result strongly suggests that a 3′ flap-like structure is involved in triggering the observed over-replication, and we hypothesized that it arises if, as part of a fork fusion reaction (Figures 5i,ii), the helicase of one replication fork complex displaces the leading strand polymerase of the opposing fork (Figure 5iii). Both template and primer are held in the active center of the replicative polymerase under tension (Joyce and Steitz, 1995; Baker and Bell, 1998; Xu and Dixon, 2018), and a ‘nudge’ of the leading strand polymerase by the DnaB helicase of the opposing fork might be enough to temporarily melt primer and template.

RecG helicase shows a particularly high affinity for 3′ flaps and processes these by converting them into a 5′ flap structure (Figure 5iv; McGlynn and Lloyd, 2001; Tanaka and Masai, 2006). In addition, 3′ flaps will also be degraded by 3′ single-stranded exonucleases, and we have shown that the absence of multiple 3′ exonucleases in particular results in high levels of over-replication in the termination area (Rudolph et al., 2010, 2013; Midgley-Smith et al., 2019). Thus, we hypothesize that the absence of either RecG or 3′ exonucleases results in the persistence of 3′ flap-like structures, a substrate for PriA, even though the affinity of PriA for a 3′ flap is lower than that of RecG (Tanaka and Masai, 2006). PriA processing will result in the assembly of a new replication fork, the progression of which will not only result in chromosomal over-replication, but also the formation of a double-stranded DNA end (Figure 5v). dsDNA ends will be rapidly processed by the homologous recombination proteins RecBCD and RecA, leading to the formation of a displacement loop (D-loop) (Rudolph et al., 2009, 2013, 2019; Dimude et al., 2016; Midgley-Smith et al., 2019). D-loops are yet another substrate for PriA, which means an opportunity for the formation of more replication forks, thus setting up a positive feedback loop of over-replication (Figure 5vi).

What data support the idea that over-replication is triggered at termination inter mediates generated by freely-fusing forks, and not at structures such as replisomes blocked by Tus-*ter* complexes? The latter cannot be the case, because over-replication was initially found in cells lacking Tus protein (Markovitz, 2005) and several recent studies have confirmed the presence of over-replication in *Δtus* cells (Rudolph et al., 2013; Dimude et al., 2015; Midgley-Smith et al., 2018, 2019). Thus, the synthesis observed is triggered independently of the fork trap system, very likely directly at fusing replication fork complexes.

**Figure 6:**
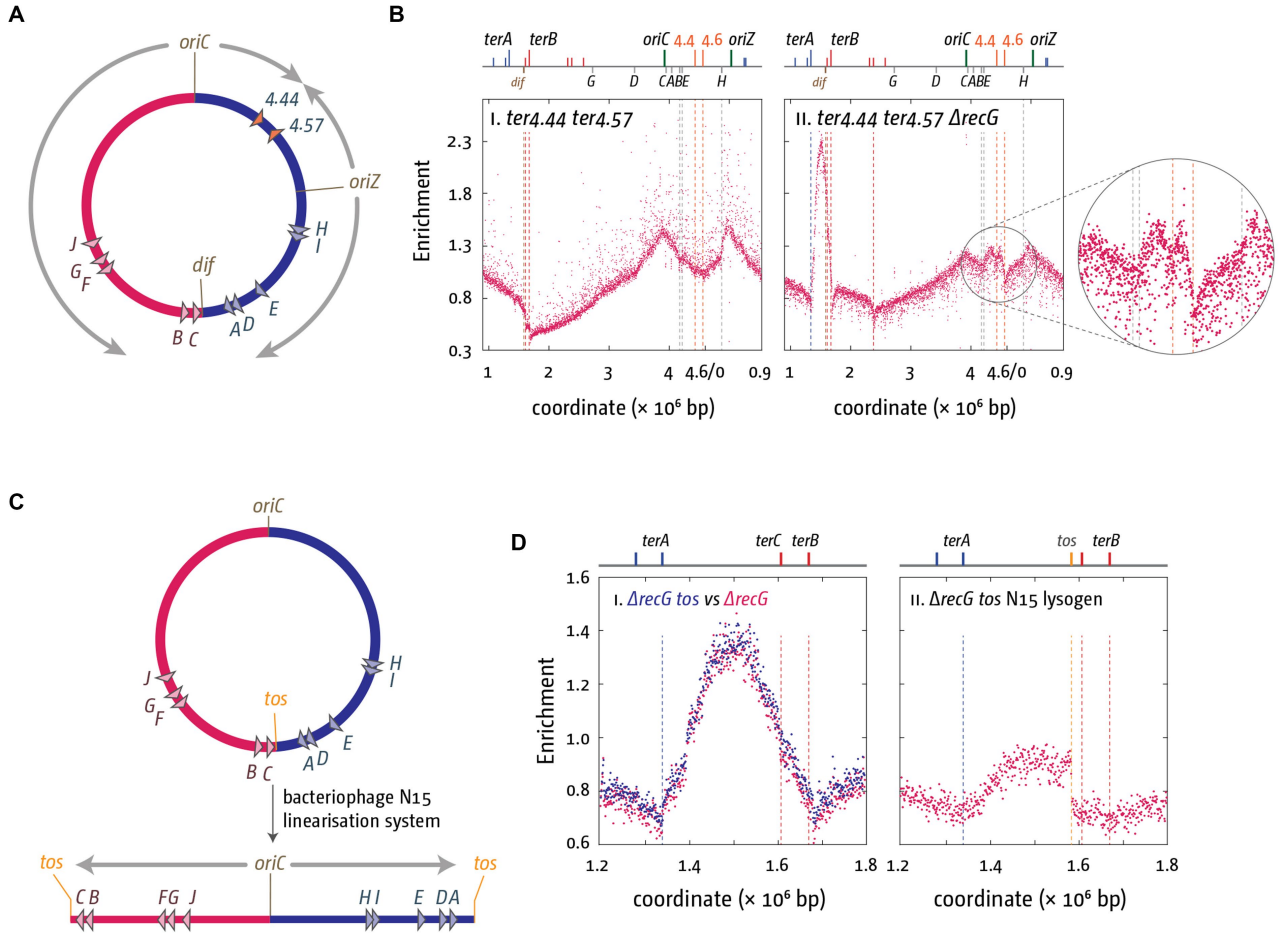
Over-replication at fork fusion sites can be modulated in *E. coli* if termination sites are artificially altered. **(A)** Schematic representation of the chromosome of *oriC^+^ oriZ^+^* cells with additional *ter* sites integrated either side of the ectopic fork fusion area. **(B)** Chromosomal marker frequency analysis (MFA) of *oriC^+^ oriZ^+^* and *oriC^+^ oriZ^+^ ΔrecG* cells with an ectopic replication fork trap in the presence and absence of RecG helicase. The numbers of reads (normalized against reads for a stationary phase wild type control) are plotted against the chromosomal location. A schematic representation of the *E. coli* chromosome showing positions of *oriC, oriZ*, native and ectopic *ter* sites (above) as well as *dif* and *rrn* operons *A–E, G*, and *H* (below) is shown above the plotted data. The data were re-plotted from Midgley-Smith et al. (2018). **(C)** Schematic representation of the linearization of the *E. coli* chromosome, as described in Cui et al. (2007) and Rudolph et al. (2013). The integrated bacteriophage N15 linearization site *tos* is highlighted. Processing by the N15 telomerase results in the formation of a linear chromosome with covalently-closed hairpin ends, which prevents forks meeting each other. **(D)** Effect of chromosome linearization on origin-independent synthesis in Δ*recG* cells. Shown is the number of reads (normalized against the reads for a stationary-phase wild-type control) plotted against the chromosomal location. In panel **(Di)** data sets for *recG* and *recG* tos are plotted together for direct comparison, while panel **(Dii)** shows the data for the linearised construct. Data replotted from Rudolph et al. (2013).

In addition, in a recent analysis we were able to show that over-replication can be triggered in the absence of RecG in defined ectopic chromosomal areas if forks are artificially forced to fuse in these areas (Midgley-Smith et al., 2018). Again, we used a strain that harbors an ectopic replication origin, *oriZ*, half-way into the right-hand replichore (Wang et al., 2011; Ivanova et al., 2015). Forks coming from *oriC* and *oriZ* fuse approximately at position 4.5 Mbp of the chromosome (Ivanova et al., 2015), and we artificially engineered an ectopic fork trap in this location, with *ter* sequences inserted at positions 4.4 and 4.6 Mbp (Figure 6A). No over-replication was observed in this location in *oriC^+^ oriZ^+^* cells in the presence of RecG, but in its absence a clear peak of over-replication was observed (Figure 6B; Midgley-Smith et al., 2018).

We also investigated what happens if replication forks are prevented from fusing. The *E. coli* chromosome can be stably linearized using the bacteriophage N15 linearization system. Cui and colleagues successfully integrated the *tos* linearization sequence into the termination area of the chromosome (Cui et al., 2007). Expression of the bacteriophage N15 telomerase resulted in the processing of the *tos* linearization sequence into two hairpin ends, which will prevent forks from meeting each other (Figure 6C; Cui et al., 2007; Rudolph et al., 2013). If fork fusion reactions are responsible for the observed over-replication, chromosome linearization should result in a significant reduction of the over-replication. This was indeed what we observed. Over-replication was significantly reduced and entirely depended on forks coming from *oriC* (Figure 6D; Rudolph et al., 2013; Dimude et al., 2015). Taken together both findings strongly support the idea that the over-replication observed in the termination area is triggered by the fusion of replication fork complexes. They are of particular importance in the light of a recent elegant study by Raghunathan and co-workers showing that in cells lacking the DNA adenine methylase, Dam, a peak of over-replication is observed in the termination area, and cells are capable of growing in the absence of a functional origin (Raghunathan et al., 2019). The authors postulate that an increased number of recombination events in all chromosomal areas triggers significant levels of recombination-dependent replication, which also results in over-replication of the termination area and, because of the fork trap, results in a peak in the marker frequency analysis (Raghunathan et al., 2019). Indeed, a role for RecG in controlling recombination-dependent replication was reported before (Azeroglu et al., 2016; Azeroglu and Leach, 2017), which might explain the small peak of synthesis remaining in *ΔrecG* cells in which the chromosome is linearized (Figure 6D). However, as recombination-dependent synthesis is triggered independently of fork fusion events, the reduction of over-replication in *ΔrecG* cells with a linear chromosome strongly suggests that it is not responsible for the significant levels of synthesis observed in the termination area in cells lacking RecG helicase.

Thus, research by our own and other labs shows that resolving the observed over-replication requires a surprising number of proteins, including RecG helicase, DNA polymerase I, the exonucleases ExoI, ExoVII, SbcCD and RecJ, the homologous recombination proteins RecBC, RecD and RuvABC and others (Markovitz, 2005; Rudolph et al., 2013; Wendel et al., 2014, 2018; Dimude et al., 2015, 2018a; Sinha et al., 2017, 2018; Midgley-Smith et al., 2018, 2019). In addition, our recent *in vitro* results suggest that more proteins are yet to be discovered (Jameson et al., 2021). As many of these intermediates arise as a direct result of fork fusions, it is tempting to speculate that they can arise generally as part of termination in bacterial DNA replication, and indeed many of the above proteins are well conserved in prokaryotes (and some beyond). Thus, the necessary proteins exist to bring termination to a successful conclusion, regardless of the presence of absence of a fork trap mechanism. However, a fork trap, once acquired, seems to add an additional layer of protection.

What are the particular benefits of a fork trap system? Bacterial cells go to great lengths to ensure that exactly one complete copy of the chromosome is generated per cell cycle (Boye et al., 2000; Skarstad and Katayama, 2013). However, if the over-replication triggered at termination intermediates is allowed to proceed, it results in uncontrolled over-replication of the chromosome, which can undermine the regulatory steps employed to control genome duplication. In cells lacking both RecG and Tus the over-replication that is triggered in the termination area can proceed into both replichores, eventually leading to dangerous head-on collisions with transcribing RNA polymerase complexes at highly transcribed genes such as *rrn* operons, as described above. Such collisions can be alleviated by so-called rpo* point mutations, point mutations in genes for some of the RNA polymerase subunits which destabilize ternary RNA polymerase complexes (Trautinger et al., 2005; Rudolph et al., 2007). If an rpo* mutation is introduced in cells lacking RecG and Tus, the synthesis in these cells is robust enough to allow cell growth and division even if the entire native origin is deleted (Rudolph et al., 2013; Dimude et al., 2015). Inhibition of *oriC* firing in ΔrecG Δtus rpo* cells revealed a replication profile that was inverted, with highest levels of synthesis in the termination area where forks normally fuse, while lowest levels were observed in the area around where *oriC* normally is located (Rudolph et al., 2013; Dimude et al., 2015). In contrast, in cells with a fork trap such over-replication is blocked by Tus-*ter* complexes (Rudolph et al., 2013; Dimude et al., 2015).

**Figure 7:**
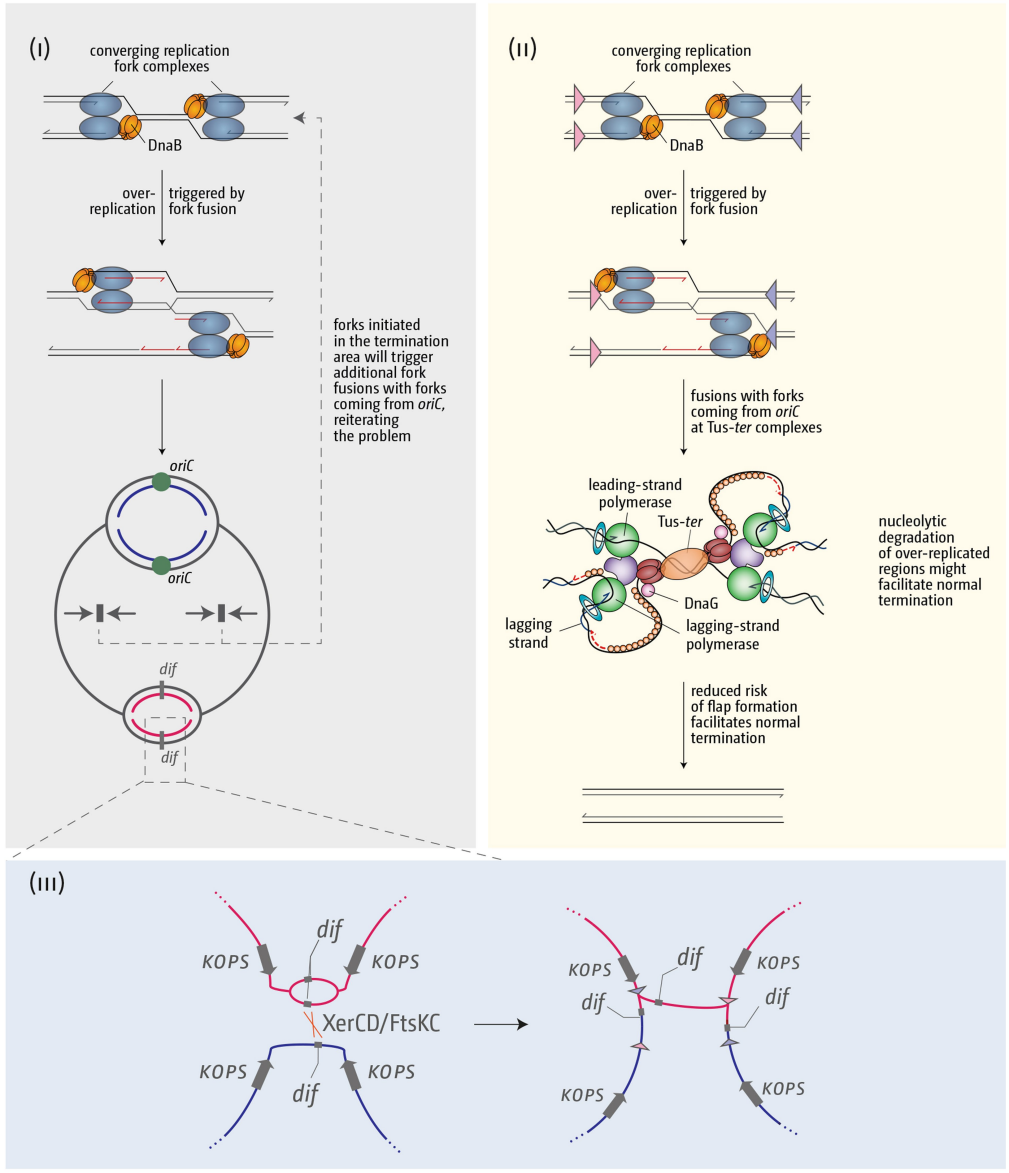
Schematic illustrating how over-replication triggered by replication fork fusions might trigger problems for chromosome duplication and segregation, and how the presence of a fork trap area might help in resolving these issues. **(i)** Over-replication triggered in the termination area by fork fusion reactions will result in additional collisions with forks coming from *oriC*, thereby reiterating and exacerbating the problem. **(ii)** A fork fusion reaction by a freely moving fork encountering a second stably arrested at a Tus-*ter* complex is less likely to result in formation of a 3’ flap and subsequent over-replication, thereby facilitating successful termination. **(iii)** If termination induces over-replication and the chromosome dimer resolution site *dif* is duplicated, this can lead to problems of the chromosome dimer resolution mechanism. See text for further details.

One danger of replication fork complexes escaping the termination area is that these forks will fuse somewhere in the replichore with forks coming from *oriC*, which poses the risk of repeating the problem (Figure 7i). In contrast, based on our genetics data we have postulated before that fork fusion events at a Tus-*ter* complex might be less prone to triggering over-replication (Midgley-Smith et al., 2019), even though additional proteins might be required to resolve replisome-Tus-*ter*-replisome super-complexes (Figure 4; Jameson et al., 2021). Thus, if replication fork complexes are stably arrested at Tus-*ter* complexes until the next round of synthesis arrives from *oriC*, the resolution of these complexes might result in successful termination (Figure 7ii). In addition, there is evidence of processing by several nucleases in the termination area, including RecBCD and SbcCD (Rudolph et al., 2013; Courcelle et al., 2015; Azeroglu et al., 2016; Sinha et al., 2017, 2018; Dimude et al., 2018a). The molecular details are complex and will depend on the precise intermediates that are being formed, but if over-replication is blocked relatively quickly, degradation could contribute to simply resolving that situation and achieve successful termination (Figure 7ii), a situation that is much harder to achieve if over-replication is allowed to proceed into the replichore in a direction opposite to normal.

#### Fork trap systems have multiple benefits

An additional benefit of a fork trap system was recently observed by Hamilton and colleagues. They measured DNA synthesis induced by χ sites, which switch the functionality of RecBCD from dsDNA degradation to the formation of a 3′ ssDNA overhang that will be bound by RecA and which can engage in recombination-dependent replication. By using a plasmid-based system the authors were able to show that the addition of *ter* sites successfully limited the amount of replication induced by χ sites, which helped to improve plasmid stability (Hamilton et al., 2023).

Furthermore, any process that involves processive translocation of large molecular machines along DNA will trigger superhelical stress (Chedin and Benham, 2020). This will be particularly problematic if the overall number of replication forks is increased by over-replication forks escaping the termination area. Superhelical stress can result in increased R-loop formation, as R-loops can help to absorb negative superhelicity (Chedin and Benham, 2020). However, increased levels of R-loops can also threaten genomic integrity (Crossley et al., 2019).

In addition, processing of fork fusion intermediates by homologous recombination proteins will increase the frequency of recombination events in the termination area, and elevated levels of recombination were indeed reported for this area in *E. coli* (Horiuchi et al., 1994). Aspects such as over-replication of already replicated DNA, increased levels of R-loops and increased recombination frequencies have all been found to contribute to genomic instability (Finkel et al., 2007; Blow and Gillespie, 2008; Alexander and Orr-Weaver, 2016; Tomasetti et al., 2017; Crossley et al., 2019), highlighting the benefits of a replication fork trap system and adding to the explanation of why fork trap systems, once acquired, are so consistently maintained.

**Figure 8:**
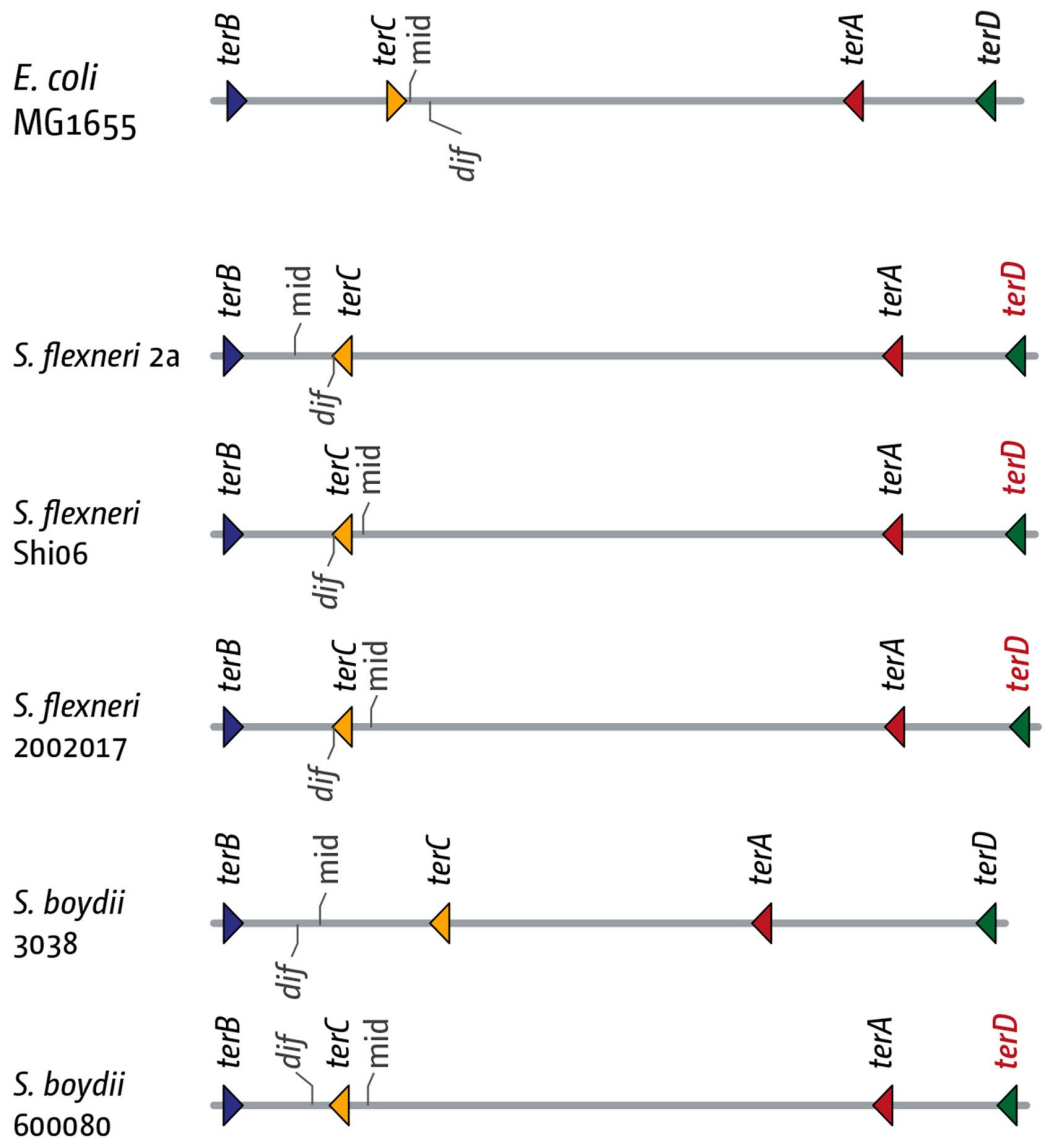
Details of the inner replication fork trap architecture in genomes from three *Shigella flexneri* and two *Shigella boydii* genomes. The inner *ter* sites and the chromosome dimer resolution site *dif* are marked. The orientation of the *ter* sites are indicated by the direction of the triangle (forks encountering the tip of the triangle would get blocked). The orientation of the *dif* chromosome dimer resolution site is indicated by the marker pointing upwards (indicating the (+)-strand) or downwards (indicating the (−)-strand). The *terD* sites highlighted in red indicate a change of the G6 residue, which makes this *ter* site much less efficient at blocking a progressing replication fork.

Another aspect where the fork trap area might play an important role is the chromosome dimer resolution site *dif*. Chromosomal dimers can form as a consequence of an odd number of recombination events. FtsK translocase, directed by FtsK-Orienting Polar Sequences (KOPS) towards the termination area and *dif*, controls a site-specific recombination system that resolves chromosomal dimers, which involves the site-specific recombinase XerCD and the *dif* site (Bigot, 2005; Levy et al., 2005; Barre, 2007; Sherratt et al., 2010). In our analysis of the different *E. coli* phylogroups we found that *dif* is consistently located between the innermost *ter* sites *A* and *C* (Figure 8). This is also the case for a variety of other bacteria with a fork trap, including *Salmonella enterica*, *Klebsiella variicola* and *Klebsiella pneumoniae*, *Edwardsiella tarda, Cedecea neteri* and *Dickeya paradisiaca* (Goodall et al., 2021; Toft et al., 2022). It is likely that this defined location of the *dif* site reflects biological importance. For the successful resolution of chromosomal dimers exactly two *dif* sites of a dimer have to be brought together, followed by XerCD processing (Castillo et al., 2017). If more than two *dif* sites are present, for example because one stretch containing a *dif* site is duplicated *via* termination-induced over-replication, this will be problematic, because a recombination event between the wrong *dif* sites will not resolve the chromosomal dimer (Figure 7iii; Goodall et al., 2021). Similarly, if two *dif* sites are available for processing before replication is complete, which might be the case if the fork fusion point is located in a different area of the chromosome to *dif*, this will be problematic, as it could trigger unwanted processing events. Indeed, we showed recently that the doubling time of a strain in which the *dif* site is moved approximately 100 kb out of the inner termination area is significantly longer (27 *vs.* 22 min) (Goodall et al., 2021). The importance of limiting the number of available *dif* sites is also highlighted by the fact that in *Vibrio cholerae*, which carries two circular chromosome, the *dif* sites of the different chromosomes have particular sequence changes, thereby avoiding inter-chromosomal attempts of processing (Castillo et al., 2017).

In this context we stumbled across an intriguing detail in *Shigella*. When analyzing the termination area in *Shigella flexneri* and *Shigella boydii* strains, we found that, in the majority of genomes investigated, the *dif* site was located between *ter* sites *B* and *C*, rather than *A* and *C* (Goodall et al., 2021). This change was accompanied with a consistent inversion of *terC*, as well as a mutation of the critical C6 base in *terD* (Figure 7). In other words, in these strains the innermost termination area is flanked by *terB* on one side, and *ter* sites *C*, *A* and *D* on the other, with *D* often inactivated, as the critical C6 is mutated (Kamada et al., 1996; Neylon et al., 2005). Thus, it seems that *dif* moves with the location of the inner termination area, or vice versa (Goodall et al., 2021). In contrast, in the absence of a replication fork trap the delay of one fork will result in progression of the opposite fork, leading to the duplication of the *dif* site before chromosome duplication is complete, generating intermediates that are not as efficiently processed at the late stages of the duplication process. Thus, by restricting the continuation of synthesis, a fork trap system is likely to alleviate all of these problems.

### Conclusion

The chromosomal architecture in bacteria is quite remarkable: the strict limitation to a single replication origin (Gao and Zhang, 2008; Gao, 2015) dictates that the speed of replication directly influences growth. Faster growth will require faster replication fork speeds, and under fast growth conditions DNA replication in *E. coli* is indeed almost 15 × faster than DNA synthesis in eukaryotic cells (Pham et al., 2013). To speed up genome duplication, bacteria such as *E. coli* use overlapping rounds of replication, while the seemingly simple solution of multiple origins is not used. Both in archaea and eukaryotic cells the use of multiple origins is the norm (Early et al., 2004; Ausiannikava and Allers, 2017). What difference allows the use of multiple origins? Eukaryotic cells have much larger genomes, the duplication of which is facilitated by an increased number of replication forks (Leonard and Méchali, 2013), but this is not a satisfactory explanation, as archaeal chromosomes and also the chromosomes of, e.g., *Saccharomyces cerevisiae* are of similar size or even smaller than the *E. coli* chromosome (Belda et al., 2019) and still several origins are present.

Multiple factors will have contributed to the development of these distinct chromosomal architectures. However, as highlighted above, termination of DNA replication can trigger the formation of intermediates that cause a variety of downstream problems. These problems include chromosomal over-replication, threatening the control that restricts genome duplication to once per cell cycle, localized increases in recombination which will destabilize the genome, topological problems, which may result in secondary effects, such as increased levels of R-loops, and problems for the processes associated with the late stages of genome duplication, such as decatenation and chromosome dimer resolution. Given the large number of possible problems, it is perhaps not surprising that a large number of conserved protein activities are reported to be involved in the rapid and effective processing of fork fusion intermediates. However, it is tempting to speculate that one easy and efficient way to restrict the number of fork fusion events is by restricting the number of origins to its minimum – a single event. We believe that a fork trap system, while not essential, adds an additional mechanistic layer to the coordination of fork movement. It enables intermediates that arise in a defined area of the chromosome to be trapped, which facilitates their processing, as well as the coordination of termination with concatenation and chromosome dimer resolution (Figure 7), explaining why, once acquired, it was so consistently retained.

But why is the increased number of origins less of a problem in archaea and eukaryotic cells? Our data strongly suggest that 3′ flaps are an important termination intermediate as replication fork complexes fuse in bacteria (Rudolph et al., 2010, 2013; Dimude et al., 2015; Midgley-Smith et al., 2019). 3′ flap formation is driven by the replicative helicase DnaB encircling the lagging strand template (LeBowitz and McMacken, 1986). In contrast, the archaeal and eukaryotic replisomes use CMG helicase to unwind the parental duplex, which will not generate a 3′ flap structure, as it is not encircling the lagging strand template (Abid Ali and Costa, 2016), which makes it likely that the precise intermediates that are generated as forks fuse differ significantly from the situation in bacteria.

However, this does not mean that the termination stage of DNA replication is unproblematic in eukaryotic cells. On the contrary, recent work by numerous labs has highlighted that the fusion of two forks needs to be carefully orchestrated as well. Similar to bacterial helicases such as RecG, the helicases Pif1 and Rrm3 play an important role in facilitating fork fusions and bringing the replication process to a successful conclusion (Steinacher et al., 2012; Deegan et al., 2019), even though replisomes appear not to slow as they move towards each other (Dewar et al., 2015; Dewar and Walter, 2017). Furthermore, upon completion of synthesis the disassembly of the replisome components needs to be carefully coordinated (Maric et al., 2014; Moreno et al., 2014; Moreno and Gambus, 2015; Sonneville et al., 2017; Zhou et al., 2021).

We are only beginning to understand the molecular mechanisms that are at play during this fascinating stage of the replication process. Future mechanistic studies in bacteria, archaea and eukaryotic cells will without doubt provide additional important mechanistic details of how replication is concluded efficiently and with high accuracy.

## Author contributions

DG and CR wrote the initial draft of the article. DG, DW, MH, and CR were involved in extensive edits of the entire text. All authors contributed to the article and approved the submitted version.

## Funding

The work was supported by Research Grant BB/N014995/1 from the Biotechnology and Biological Sciences Research Council to CR and MH, and Research Grant BB/W000393/1 from the Biotechnology and Biological Sciences Research Council to CR.

## Conflict of interest

The authors declare that the research was conducted in the absence of any commercial or financial relationships that could be construed as a potential conflict of interest.

## Publisher’s note

All claims expressed in this article are solely those of the authors and do not necessarily represent those of their affiliated organizations, or those of the publisher, the editors and the reviewers. Any product that may be evaluated in this article, or claim that may be made by its manufacturer, is not guaranteed or endorsed by the publisher.

## References

[ref1] Abhyankar M. M. Reddy J. M. Sharma R. Büllesbach E. Bastia D. (2004). Biochemical investigations of control of replication initiation of plasmid R6K. J. Biol. Chem. 279, 6711–6719. doi: 10.1074/jbc.M312052200 14665626

[ref2] Abhyankar M. M. Zzaman S. Bastia D. (2003). Reconstitution of R6K DNA replication in vitro using 22 purified proteins. J. Biol. Chem. 278, 45476–45484. doi: 10.1074/jbc.M308516200, PMID: 12970346

[ref3] Abid Ali F. Costa A. (2016). The MCM helicase motor of the eukaryotic replisome. J. Mol. Biol. 428, 1822–1832. doi: 10.1016/j.jmb.2016.01.024 26829220

[ref4] Ahn K. S. Malo M. S. Smith M. T. Wake R. G. (1993). Autoregulation of the gene encoding the replication terminator protein of *Bacillus subtilis*. Gene 132, 7–13. doi: 10.1016/0378-1119(93)90508-Z 8406044

[ref5] Alexander J. L. Orr-Weaver T. L. (2016). Replication fork instability and the consequences of fork collisions from rereplication. Genes Dev. 30, 2241–2252. doi: 10.1101/gad.288142.116 27898391 PMC5110991

[ref6] Ausiannikava D. Allers T. (2017). Diversity of DNA replication in the Archaea. Genes 8:E56. doi: 10.3390/genes8020056 PMC5333045 28146124

[ref7] Azeroglu B. Leach D. R. F. (2017). RecG controls DNA amplification at double-strand breaks and arrested replication forks. FEBS Lett. 591, 1101–1113. doi: 10.1002/1873-3468.12583 28155219 PMC5412681

[ref8] Azeroglu B. Mawer J. S. P. Cockram C. A. White M. A. Hasan A. M. M. Filatenkova M.. (2016). RecG directs DNA synthesis during double-Strand break repair. PLoS Genet. 12:e1005799. doi: 10.1371/journal.pgen.1005799, PMID: 26872352 PMC4752480

[ref9] Baker T. A. Bell S. P. (1998). Polymerases and the replisome: machines within machines. Cells 92, 295–305. doi: 10.1016/S0092-8674(00)80923-X 9476890

[ref10] Barre F.-X. (2007). FtsK and SpoIIIE: the tale of the conserved tails. Mol. Microbiol. 66, 1051–1055. doi: 10.1111/j.1365-2958.2007.05981.x 17973909

[ref11] Bastia D. Zzaman S. Krings G. Saxena M. Peng X. Greenberg M. M. (2008). Replication termination mechanism as revealed by Tus-mediated polar arrest of a sliding helicase. Proc. Natl. Acad. Sci. U. S. A. 105, 12831–12836. doi: 10.1073/pnas.0805898105, PMID: 18708526 PMC2529109

[ref12] Belda I. Ruiz J. Santos A. Wyk N. V. Pretorius I. S. (2019). Saccharomyces cerevisiae. Trends Genet. 35, 956–957. doi: 10.1016/j.tig.2019.08.009 31630852

[ref13] Berghuis B. A. Dulin D. Xu Z.-Q. van Laar T. Cross B. Janissen R.. (2015). Strand separation establishes a sustained lock at the Tus-Ter replication fork barrier. Nat. Chem. Biol. 11, 579–585. doi: 10.1038/nchembio.1857 26147356

[ref14] Berghuis B. A. Raducanu V.-S. Elshenawy M. M. Jergic S. Depken M. Dixon N. E.. (2018). What is all this fuss about Tus? Comparison of recent findings from biophysical and biochemical experiments. Crit. Rev. Biochem. Mol. Biol. 53, 49–63. doi: 10.1080/10409238.2017.1394264 29108427

[ref15] Bianco P. R. (2020). DNA helicase-SSB interactions critical to the regression and restart of stalled DNA replication forks in *Escherichia coli*. Genes 11:471. doi: 10.3390/genes11050471 32357475 PMC7290993

[ref16] Bidnenko V. Ehrlich S. D. Michel B. (2002). Replication fork collapse at replication terminator sequences. EMBO J. 21, 3898–3907. doi: 10.1093/emboj/cdf369, PMID: 12110601 PMC126115

[ref17] Bidnenko V. Lestini R. Michel B. (2006). The *Escherichia coli* UvrD helicase is essential for Tus removal during recombination-dependent replication restart from Ter sites. Mol. Microbiol. 62, 382–396. doi: 10.1111/j.1365-2958.2006.05382.x 17020578

[ref18] Bigot (2005). KOPS: DNA motifs that control E-coli chromosome segregation by orienting the FtsK translocase. EMBO J. 24, 3770–3780. doi: 10.1038/sj.emboj.7600835 16211009 PMC1276719

[ref19] Bird R. E. Louarn J. Martuscelli J. Caro L. (1972). Origin and sequence of chromosome replication in *Escherichia coli*. J. Mol. Biol. 70, 549–566. doi: 10.1016/0022-2836(72)90559-1 4563262

[ref20] Blattner F. R. Plunkett G. Bloch C. A. Perna N. T. Burland V. Riley M.. (1997). The complete genome sequence of *Escherichia coli* K-12. Science 277, 1453–1462. doi: 10.1126/science.277.5331.1453 9278503

[ref21] Blow J. J. Ge X. Q. Jackson D. A. (2011). How dormant origins promote complete genome replication. Trends Biochem. Sci. 36, 405–414. doi: 10.1016/j.tibs.2011.05.002 21641805 PMC3329722

[ref22] Blow J. J. Gillespie P. J. (2008). Replication licensing and cancer--a fatal entanglement? Nat. Rev. Cancer 8, 799–806. doi: 10.1038/nrc2500 18756287 PMC2577763

[ref23] Boubakri H. de Septenville A. L. Viguera E. Michel B. (2010). The helicases DinG, rep and UvrD cooperate to promote replication across transcription units in vivo. EMBO J. 29, 145–157. doi: 10.1038/emboj.2009.308 19851282 PMC2770101

[ref24] Bouché J. P. Gélugne J. P. Louarn J. Louarn J. M. Kaiser K. (1982). Relationships between the physical and genetic maps of a 470 x 10(3) base-pair region around the terminus of *Escherichia coli* K12 DNA replication. J. Mol. Biol. 154, 21–32. 6281437 10.1016/0022-2836(82)90414-4

[ref25] Boye E. Løbner-Olesen A. Skarstad K. (2000). Limiting DNA replication to once and only once. EMBO Rep. 1, 479–483. doi: 10.1093/embo-reports/kvd116 11263490 PMC1083788

[ref26] Brewer B. J. (1988). When polymerases collide: replication and the transcriptional organization of the *E. coli* chromosome. Cells 53, 679–686. 10.1016/0092-8674(88)90086-4 3286014

[ref27] Cao J. Woodhall M. R. Alvarez J. Cartron M. L. Andrews S. C. (2007). EfeUOB (YcdNOB) is a tripartite, acid-induced and CpxAR-regulated, low-pH Fe2+ transporter that is cryptic in *Escherichia coli* K-12 but functional in *E. coli* O157:H7. Mol. Microbiol. 65, 857–875. doi: 10.1111/j.1365-2958.2007.05802.x 17627767

[ref28] Castillo F. Benmohamed A. Szatmari G. (2017). Xer site specific recombination: double and single recombinase systems. Front. Microbiol. 8:453. doi: 10.3389/fmicb.2017.00453, PMID: 28373867 PMC5357621

[ref29] Chedin F. Benham C. J. (2020). Emerging roles for R-loop structures in the management of topological stress. J. Biol. Chem. 295, 4684–4695. doi: 10.1074/jbc.REV119.006364, PMID: 32107311 PMC7135976

[ref30] Courcelle J. Wendel B. M. Livingstone D. D. Courcelle C. T. (2015). RecBCD is required to complete chromosomal replication: implications for double-strand break frequencies and repair mechanisms. DNA Repair 32, 86–95. doi: 10.1016/j.dnarep.2015.04.018 26003632 PMC4522357

[ref31] Crosa J. H. Luttropp L. K. Falkow S. (1976). Mode of replication of the conjugative R-plasmid RSF1040 in *Escherichia coli*. J. Bacteriol. 126, 454–466. 770431 10.1128/jb.126.1.454-466.1976 PMC233302

[ref32] Crossley M. P. Bocek M. Cimprich K. A. (2019). R-loops as cellular regulators and genomic threats. Mol. Cell 73, 398–411. doi: 10.1016/j.molcel.2019.01.024 30735654 PMC6402819

[ref33] Cui T. Moro-oka N. Ohsumi K. Kodama K. Ohshima T. Ogasawara N.. (2007). *Escherichia coli* with a linear genome. EMBO Rep. 8, 181–187. doi: 10.1038/sj.embor.7400880 17218953 PMC1796773

[ref34] de Graaff J. Crosa J. H. Heffron F. Falkow S. (1978). Replication of the nonconjugative plasmid RSF1010 in *Escherichia coli* K-12. J. Bacteriol. 134, 1117–1122. doi: 10.1128/jb.134.3.1117-1122.1978, PMID: 350840 PMC222362

[ref35] de Massy B. Béjar S. Louarn J. Louarn J. M. Bouché J. P. (1987). Inhibition of replication forks exiting the terminus region of the *Escherichia coli* chromosome occurs at two loci separated by 5 min. Proc. Natl. Acad. Sci. U. S. A. 84, 1759–1763. 3550797 10.1073/pnas.84.7.1759 PMC304520

[ref36] De Septenville A. L. Duigou S. Boubakri H. Michel B. (2012). Replication fork reversal after replication-transcription collision. PLoS Genet. 8:e1002622. doi: 10.1371/journal.pgen.1002622 22496668 PMC3320595

[ref37] Deegan T. D. Baxter J. Ortiz Bazán M. Á. Yeeles J. T. P. Labib K. P. M. (2019). Pif1-family helicases support fork convergence during DNA replication termination in eukaryotes. Mol. Cell 74, 231–244.e9. doi: 10.1016/j.molcel.2019.01.040 30850330 PMC6477153

[ref38] Dewar J. M. Budzowska M. Walter J. C. (2015). The mechanism of DNA replication termination in vertebrates. Nature 525, 345–350. doi: 10.1038/nature14887, PMID: 26322582 PMC4575634

[ref39] Dewar J. M. Walter J. C. (2017). Mechanisms of DNA replication termination. Nat. Rev. Mol. Cell Biol. 18, 507–516. doi: 10.1038/nrm.2017.42 28537574 PMC6386472

[ref40] Dimude J. U. Midgley-Smith S. L. Rudolph C. J. (2018a). Replication-transcription conflicts trigger extensive DNA degradation in *Escherichia coli* cells lacking RecBCD. DNA Repair 70, 37–48. doi: 10.1016/j.dnarep.2018.08.002 30145455

[ref41] Dimude J. U. Midgley-Smith S. L. Stein M. Rudolph C. J. (2016). Replication termination: containing fork fusion-mediated pathologies in *Escherichia coli*. Genes 7:40. doi: 10.3390/genes7080040 27463728 PMC4999828

[ref42] Dimude J. U. Stein M. Andrzejewska E. E. Khalifa M. S. Gajdosova A. Retkute R.. (2018b). Origins left, right, and Centre: increasing the number of initiation sites in the *Escherichia coli* chromosome. Genes 9:376. doi: 10.3390/genes9080376 30060465 PMC6116050

[ref43] Dimude J. U. Stockum A. Midgley-Smith S. L. Upton A. L. Foster H. A. Khan A.. (2015). The consequences of replicating in the wrong orientation: bacterial chromosome duplication without an active replication origin. mBio:6. doi: 10.1128/mBio.01294-15 PMC4631800 26530381

[ref44] Duggin I. G. Bell S. D. (2009). Termination structures in the *Escherichia coli* chromosome replication fork trap. J. Mol. Biol. 387, 532–539. doi: 10.1016/j.jmb.2009.02.027, PMID: 19233209

[ref45] Duggin I. G. Wake R. G. Bell S. D. Hill T. M. (2008). The replication fork trap and termination of chromosome replication. Mol. Microbiol. 70, 1323–1333. doi: 10.1111/j.1365-2958.2008.06500.x 19019156

[ref46] Early A. Drury L. Diffley J. F. X. (2004). Mechanisms involved in regulating DNA replication origins during the cell cycle and in response to DNA damage. Philos. Trans. R. Soc. Lond. Ser. B Biol. Sci. 359, 31–38. doi: 10.1098/rstb.2003.1362, PMID: 15065654 PMC1693309

[ref47] Eli Bussiere D. Bastia D. Stephen White W. (1995). Crystal structure of the replication terminator protein from *B. subtilis* at 2.6 Å. Cells 80, 651–660. doi: 10.1016/0092-8674(95)90519-7 7867072

[ref48] Elshenawy M. M. Jergic S. Xu Z.-Q. Sobhy M. A. Takahashi M. Oakley A. J.. (2015). Replisome speed determines the efficiency of the Tus-Ter replication termination barrier. Nature 525, 394–398. doi: 10.1038/nature14866 26322585

[ref49] Finkel T. Serrano M. Blasco M. A. (2007). The common biology of cancer and ageing. Nature 448, 767–774. doi: 10.1038/nature05985 17700693

[ref50] François V. Louam J. Louarn J.-M. (1989). The terminus of the *Escherichia coli* chromosome is flanked by several polar polar replication pause sites. Mol. Microbiol. 3, 995–1002. doi: 10.1111/j.1365-2958.1989.tb00250.x 2532703

[ref51] Franks A. Griffiths A. A. Wake R. G. (1995). Identification and characterization of new DNA replication terminators in *Bacillus subtilis*. Mol. Microbiol. 17, 13–23. doi: 10.1111/j.1365-2958.1995.mmi_17010013.x 7476199

[ref52] Franks A. H. Wake R. G. (1996). Replication fork arrest at relocated replication terminators on the *Bacillus subtilis* chromosome. J. Bacteriol. 178, 4258–4265. doi: 10.1128/jb.178.14.4258-4265.1996 8763955 PMC178184

[ref53] Galli E. Ferat J.-L. Desfontaines J.-M. Val M.-E. Skovgaard O. Barre F.-X.. (2019). Replication termination without a replication fork trap. Sci. Rep. 9:8315. doi: 10.1038/s41598-019-43795-2 31165739 PMC6549158

[ref54] Gao F. (2015). Bacteria may have multiple replication origins. Front. Microbiol. 6:324. doi: 10.3389/fmicb.2015.00324, PMID: 25941523 PMC4403523

[ref55] Gao F. Zhang C.-T. (2008). Ori-finder: A web-based system for finding oriC s in unannotated bacterial genomes. BMC Bioinformatics 9:79. doi: 10.1186/1471-2105-9-79 18237442 PMC2275245

[ref56] Goodall D. J. Jameson K. H. Hawkins M. Rudolph C. J. (2021). A fork trap in the chromosomal termination area is highly conserved across all *Escherichia coli* phylogenetic groups. Int. J. Mol. Sci. 22:7928. doi: 10.3390/ijms22157928 34360694 PMC8347550

[ref57] Gottlieb P. A. Wu S. Zhang X. Tecklenburg M. Kuempel P. Hill T. M. (1992). Equilibrium, kinetic, and footprinting studies of the Tus-Ter protein-DNA interaction. J. Biol. Chem. 267, 7434–7443. 1313800

[ref58] Gregg A. V. McGlynn P. Jaktaji R. P. Lloyd R. G. (2002). Direct rescue of stalled DNA replication forks via the combined action of PriA and RecG helicase activities. Mol. Cell 9, 241–251. doi: 10.1016/s1097-2765(02)00455-0 11864599

[ref59] Griffiths A. A. Andersen P. A. Wake R. G. (1998). Replication terminator protein-based replication fork-arrest systems in various *Bacillus* species. J. Bacteriol. 180, 3360–3367. doi: 10.1128/JB.180.13.3360-3367.1998, PMID: 9642188 PMC107290

[ref60] Grosse C. Scherer J. Koch D. Otto M. Taudte N. Grass G. (2006). A new ferrous iron-uptake transporter, EfeU (YcdN), from *Escherichia coli*. Mol. Microbiol. 62, 120–131. doi: 10.1111/j.1365-2958.2006.05326.x 16987175

[ref61] Gyurasits E. B. Wake R. G. (1973). Bidirectional chromosome replication in *Bacillus subtilis*. J. Mol. Biol. 73, 55–63. doi: 10.1016/0022-2836(73)90158-7 4632203

[ref62] Hamilton N. A. Jehru A. E. Samples W. N. Wendel B. M. Mokhtari P. D. Courcelle C. T.. (2023). Chi sequences switch the RecBCD helicase-nuclease complex from degradative to replicative modes during the completion of DNA replication. J. Biol. Chem. 299:103013. doi: 10.1016/j.jbc.2023.103013, PMID: 36781123 PMC10025158

[ref63] Hansen F. G. Atlung T. (2018). The DnaA tale. Front. Microbiol. 9:319. doi: 10.3389/fmicb.2018.00319 29541066 PMC5835720

[ref64] Hasebe T. Narita K. Hidaka S. Su’etsugu M. (2018). Efficient arrangement of the replication fork trap for in vitro propagation of monomeric circular DNA in the chromosome-replication cycle reaction. Life Basel Switz. 8:43. doi: 10.3390/life8040043 PMC6315707 30257439

[ref65] Hawkins M. Dimude J. U. Howard J. A. L. Smith A. J. Dillingham M. S. Savery N. J.. (2019). Direct removal of RNA polymerase barriers to replication by accessory replicative helicases. Nucleic Acids Res. 47, 5100–5113. doi: 10.1093/nar/gkz170 30869136 PMC6547429

[ref66] Hendrickson H. Lawrence J. G. (2007). Mutational bias suggests that replication termination occurs near the dif site, not at Ter sites. Mol. Microbiol. 64, 42–56. doi: 10.1111/j.1365-2958.2007.05596.x 17376071

[ref67] Hiasa H. Marians K. J. (1994). Tus prevents overreplication of oriC plasmid DNA. J. Biol. Chem. 269, 26959–26968. 7929435

[ref68] Hidaka M. Kobayashi T. Horiuchi T. (1991). A newly identified DNA replication terminus site, TerE, on the *Escherichia coli* chromosome. J. Bacteriol. 173, 391–393. 1824765 10.1128/jb.173.1.391-393.1991 PMC207198

[ref70] Hill T. M. Henson J. M. Kuempel P. L. (1987). The terminus region of the *Escherichia coli* chromosome contains two separate loci that exhibit polar inhibition of replication. Proc. Natl. Acad. Sci. U. S. A. 84, 1754–1758. doi: 10.1073/pnas.84.7.1754, PMID: 3550796 PMC304519

[ref71] Hill T. M. Kopp B. J. Kuempel P. L. (1988a). Termination of DNA replication in *Escherichia coli* requires a trans-acting factor. J. Bacteriol. 170, 662–668. 3276664 10.1128/jb.170.2.662-668.1988 PMC210706

[ref72] Hill T. M. Marians K. J. (1990). *Escherichia coli* Tus protein acts to arrest the progression of DNA replication forks in vitro. Proc. Natl. Acad. Sci. U. S. A. 87, 2481–2485. 2181438 10.1073/pnas.87.7.2481 PMC53713

[ref73] Hill T. M. Pelletier A. J. Tecklenburg M. L. Kuempel P. L. (1988b). Identification of the DNA sequence from the *E. coli* terminus region that halts replication forks. Cells 55, 459–466. 10.1016/0092-8674(88)90032-3 2846183

[ref74] Hill T. M. Tecklenburg M. L. Pelletier A. J. Kuempel P. L. (1989). Tus, the trans-acting gene required for termination of DNA replication in *Escherichia coli*, encodes a DNA-binding protein. Proc. Natl. Acad. Sci. U. S. A. 86, 1593–1597. doi: 10.1073/pnas.86.5.1593 2646639 PMC286744

[ref75] Hizume K. Araki H. (2019). Replication fork pausing at protein barriers on chromosomes. FEBS Lett. 593, 1449–1458. doi: 10.1002/1873-3468.13481 31199500

[ref76] Horiuchi T. Fujimura Y. Nishitani H. Kobayashi T. Hidaka M. (1994). The DNA replication fork blocked at the Ter site may be an entrance for the RecBCD enzyme into duplex DNA. J. Bacteriol. 176, 4656–4663. 8045897 10.1128/jb.176.15.4656-4663.1994 PMC196287

[ref77] Hyrien O. (2000). Mechanisms and consequences of replication fork arrest. Biochimie 82, 5–17. 10717381 10.1016/s0300-9084(00)00344-8

[ref78] Ivanova D. Taylor T. Smith S. L. Dimude J. U. Upton A. L. Mehrjouy M. M.. (2015). Shaping the landscape of the *Escherichia coli* chromosome: replication-transcription encounters in cells with an ectopic replication origin. Nucleic Acids Res. 43, 7865–7877. doi: 10.1093/nar/gkv704 26160884 PMC4652752

[ref79] Jameson K. H. Rudolph C. J. Hawkins M. (2021). Termination of DNA replication at Tus-ter barriers results in under-replication of template DNA. J. Biol. Chem. 297:101409. doi: 10.1016/j.jbc.2021.101409 34780717 PMC8661018

[ref80] Jameson K. H. Wilkinson A. J. (2017). Control of initiation of DNA replication in Bacillus subtilis and *Escherichia coli*. Genes 8:22. doi: 10.3390/genes8010022 28075389 PMC5295017

[ref81] Joyce C. M. Steitz T. A. (1995). Polymerase structures and function: variations on a theme? J. Bacteriol. 177, 6321–6329. doi: 10.1128/jb.177.22.6321-6329.1995 7592405 PMC177480

[ref82] Kamada K. Horiuchi T. Ohsumi K. Shimamoto N. Morikawa K. (1996). Structure of a replication-terminator protein complexed with DNA. Nature 383, 598–603. doi: 10.1038/383598a0 8857533

[ref83] Katayama T. Kasho K. Kawakami H. (2017). The DnaA cycle in *Escherichia coli*: activation, function and inactivation of the initiator protein. Front. Microbiol. 8:2496. doi: 10.3389/fmicb.2017.02496 29312202 PMC5742627

[ref84] Khatri G. S. MacAllister T. Sista P. R. Bastia D. (1989). The replication terminator protein of *E. coli* is a DNA sequence-specific contra-helicase. Cells 59, 667–674. doi: 10.1016/0092-8674(89)90012-3, PMID: 2684415

[ref85] Kim N. Jinks-Robertson S. (2012). Transcription as a source of genome instability. Nat. Rev. Genet. 13, 204–214. doi: 10.1038/nrg3152, PMID: 22330764 PMC3376450

[ref86] Kirby R. (2011). Chromosome diversity and similarity within the Actinomycetales. FEMS Microbiol. Lett. 319, 1–10. doi: 10.1111/j.1574-6968.2011.02242.x 21320158

[ref87] Kono N. Arakawa K. Tomita M. (2012). Validation of bacterial replication termination models using simulation of genomic mutations. PLoS One 7:e34526. doi: 10.1371/journal.pone.0034526, PMID: 22509315 PMC3317982

[ref88] Krabbe M. Zabielski J. Bernander R. Nordström K. (1997). Inactivation of the replication-termination system affects the replication mode and causes unstable maintenance of plasmid R1. Mol. Microbiol. 24, 723–735. 9194700 10.1046/j.1365-2958.1997.3791747.x

[ref89] Kuempel P. L. Duerr S. A. Seeley N. R. (1977). Terminus region of the chromosome in *Escherichia coli* inhibits replication forks. Proc. Natl. Acad. Sci. U. S. A. 74, 3927–3931. 333449 10.1073/pnas.74.9.3927 PMC431788

[ref90] Kurth I. O’Donnell M. (2009). Replisome dynamics during chromosome duplication. EcoSal Plus 3:10.1128/ecosalplus.4.4.2. doi: 10.1128/ecosalplus.4.4.2 PMC4231440 26443767

[ref91] Labib K. Hodgson B. (2007). Replication fork barriers: pausing for a break or stalling for time? EMBO Rep. 8, 346–353. doi: 10.1038/sj.embor.7400940 17401409 PMC1852754

[ref92] Lang K. S. Merrikh H. (2018). The clash of macromolecular titans: replication-transcription conflicts in Bacteria. Annu. Rev. Microbiol. 72, 71–88. doi: 10.1146/annurev-micro-090817-062514 29856930 PMC6233710

[ref93] LeBowitz J. H. McMacken R. (1986). The *Escherichia coli* dnaB replication protein is a DNA helicase. J. Biol. Chem. 261, 4738–4748. doi: 10.1016/S0021-9258(17)38564-2 3007474

[ref94] Lee E. H. Kornberg A. Hidaka M. Kobayashi T. Horiuchi T. (1989). *Escherichia coli* replication termination protein impedes the action of helicases. Proc. Natl. Acad. Sci. U. S. A. 86, 9104–9108. doi: 10.1073/pnas.86.23.9104 2556700 PMC298442

[ref95] Lemon K. P. Kurtser I. Grossman A. D. (2001). Effects of replication termination mutants on chromosome partitioning in *Bacillus subtilis*. Proc. Natl. Acad. Sci. U. S. A. 98, 212–217. doi: 10.1073/pnas.011506098, PMID: 11134515 PMC14570

[ref96] Leonard A. C. Méchali M. (2013). DNA replication origins. Cold Spring Harb. Perspect. Biol. 5:a010116. doi: 10.1101/cshperspect.a010116 23838439 PMC3783049

[ref97] Levy O. Ptacin J. L. Pease P. J. Gore J. Eisen M. B. Bustamante C.. (2005). Identification of oligonucleotide sequences that direct the movement of the *Escherichia coli* FtsK translocase. Proc. Natl. Acad. Sci. U. S. A. 102, 17618–17623. doi: 10.1073/pnas.0508932102, PMID: 16301526 PMC1287487

[ref98] Lewis P. J. Ralston G. B. Christopherson R. I. Wake R. G. (1990). Identification of the replication terminator protein binding sites in the terminus region of the *Bacillus subtilis* chromosome and stoichiometry of the binding. J. Mol. Biol. 214, 73–84. doi: 10.1016/0022-2836(90)90147-E 2115089

[ref99] Linke R. Limmer M. Juranek S. A. Heine A. Paeschke K. (2021). The relevance of G-quadruplexes for DNA repair. Int. J. Mol. Sci. 22:12599. doi: 10.3390/ijms222212599 34830478 PMC8620898

[ref100] Louarn J. Patte J. Louarn J. M. (1977). Evidence for a fixed termination site of chromosome replication in *Escherichia coli* K12. J. Mol. Biol. 115, 295–314. doi: 10.1016/0022-2836(77)90156-5, PMID: 338909

[ref101] Louarn J. Patte J. Louarn J. M. (1979). Map position of the replication terminus on the *Escherichia coli* chromosome. Mol. Gen. Genet. MGG 172, 7–11. doi: 10.1007/BF00276208 377025

[ref102] MacAllister T. Khatri G. S. Bastia D. (1990). Sequence-specific and polarized replication termination in vitro: complementation of extracts of tus- *Escherichia coli* by purified Ter protein and analysis of termination intermediates. Proc. Natl. Acad. Sci. U. S. A. 87, 2828–2832. doi: 10.1073/pnas.87.7.2828 2181452 PMC53784

[ref103] Marians K. J. (2018). Lesion bypass and the reactivation of stalled replication forks. Annu. Rev. Biochem. 87, 217–238. doi: 10.1146/annurev-biochem-062917-011921, PMID: 29298091 PMC6419508

[ref104] Maric M. Maculins T. De Piccoli G. Labib K. (2014). Cdc48 and a ubiquitin ligase drive disassembly of the CMG helicase at the end of DNA replication. Science 346:1253596. doi: 10.1126/science.1253596, PMID: 25342810 PMC4300516

[ref105] Markovitz A. (2005). A new in vivo termination function for DNA polymerase I of *Escherichia coli* K12. Mol. Microbiol. 55, 1867–1882. doi: 10.1111/j.1365-2958.2005.04513.x 15752206

[ref106] Masters M. Broda P. (1971). Evidence for the bidirectional replications of the *Escherichia coli* chromosome. Nature. New Biol. 232, 137–140. doi: 10.1038/newbio232137a0, PMID: 4937091

[ref107] McGlynn P. Lloyd R. G. (2001). Rescue of stalled replication forks by RecG: simultaneous translocation on the leading and lagging strand templates supports an active DNA unwinding model of fork reversal and Holliday junction formation. Proc. Natl. Acad. Sci. 98, 8227–8234. doi: 10.1073/pnas.111008698 11459957 PMC37425

[ref108] McGlynn P. Savery N. J. Dillingham M. S. (2012). The conflict between DNA replication and transcription. Mol. Microbiol. 85, 12–20. doi: 10.1111/j.1365-2958.2012.08102.x 22607628

[ref109] McLean M. J. Wolfe K. H. Devine K. M. (1998). Base composition skews, replication orientation, and gene orientation in 12 prokaryote genomes. J. Mol. Evol. 47, 691–696. 9847411 10.1007/pl00006428

[ref110] Médigue C. Krin E. Pascal G. Barbe V. Bernsel A. Bertin P. N.. (2005). Coping with cold: the genome of the versatile marine Antarctica bacterium *Pseudoalteromonas haloplanktis* TAC125. Genome Res. 15, 1325–1335. doi: 10.1101/gr.4126905, PMID: 16169927 PMC1240074

[ref111] Merrikh H. (2017). Spatial and temporal control of evolution through replication-transcription conflicts. Trends Microbiol. 25, 515–521. doi: 10.1016/j.tim.2017.01.008 28216294 PMC5474121

[ref112] Merrikh C. N. Merrikh H. (2018). Gene inversion potentiates bacterial evolvability and virulence. Nat. Commun. 9:4662. doi: 10.1038/s41467-018-07110-3 30405125 PMC6220195

[ref113] Merrikh C. N. Weiss E. Merrikh H. (2016). The accelerated evolution of lagging Strand genes is independent of sequence context. Genome Biol. Evol. 8, 3696–3702. doi: 10.1093/gbe/evw274 28039230 PMC5585990

[ref114] Merrikh H. Zhang Y. Grossman A. D. Wang J. D. (2012). Replication-transcription conflicts in bacteria. Nat. Rev. Microbiol. 10, 449–458. doi: 10.1038/nrmicro2800 22669220 PMC3467967

[ref115] Midgley-Smith S. L. Dimude J. U. Rudolph C. J. (2019). A role for 3′ exonucleases at the final stages of chromosome duplication in *Escherichia coli*. Nucleic Acids Res. 47, 1847–1860. doi: 10.1093/nar/gky1253 30544222 PMC6393302

[ref116] Midgley-Smith S. L. Dimude J. U. Taylor T. Forrester N. M. Upton A. L. Lloyd R. G.. (2018). Chromosomal over-replication in *Escherichia coli* recG cells is triggered by replication fork fusion and amplified if replichore symmetry is disturbed. Nucleic Acids Res. 46, 7701–7715. doi: 10.1093/nar/gky566 29982635 PMC6125675

[ref117] Moreno S. P. Bailey R. Campion N. Herron S. Gambus A. (2014). Polyubiquitylation drives replisome disassembly at the termination of DNA replication. Science 346, 477–481. doi: 10.1126/science.1253585 25342805

[ref118] Moreno S. P. Gambus A. (2015). Regulation of unperturbed DNA replication by Ubiquitylation. Genes 6, 451–468. doi: 10.3390/genes6030451, PMID: 26121093 PMC4584310

[ref119] Mulcair M. D. Schaeffer P. M. Oakley A. J. Cross H. F. Neylon C. Hill T. M.. (2006). A molecular mousetrap determines polarity of termination of DNA replication in *E. coli*. Cells 125, 1309–1319. doi: 10.1016/j.cell.2006.04.040 16814717

[ref120] Mulugu S. Potnis A. Shamsuzzaman T. Alexander K. Bastia D. (2001). Mechanism of termination of DNA replication of *Escherichia coli* involves helicase-contrahelicase interaction. Proc. Natl. Acad. Sci. U. S. A. 98, 9569–9574. doi: 10.1073/pnas.171065898, PMID: 11493686 PMC55493

[ref121] Nakabachi A. Yamashita A. Toh H. Ishikawa H. Dunbar H. E. Moran N. A.. (2006). The 160-kilobase genome of the bacterial endosymbiont Carsonella. Science 314:267. doi: 10.1126/science.1134196 17038615

[ref122] Natarajan S. Kelley W. L. Bastia D. (1991). Replication terminator protein of *Escherichia coli* is a transcriptional repressor of its own synthesis. Proc. Natl. Acad. Sci. U. S. A. 88, 3867–3871. 2023933 10.1073/pnas.88.9.3867 PMC51554

[ref69] Neidhardt F. C. Curtiss R. Ingraham J. L. Lin E. C. C. Low K. B. Magasanik B.. (eds). (1996). Escherichia coli and Salmonella: cellular and molecular biology, 2nd ed. Washington, D.C.: ASM Press.

[ref123] Nester E. W. (2015). Agrobacterium: nature’s genetic engineer. Front. Plant Sci. 5:730. doi: 10.3389/fpls.2014.00730, PMID: 25610442 PMC4285021

[ref124] Neylon C. Brown S. E. Kralicek A. V. Miles C. S. Love C. A. Dixon N. E. (2000). Interaction of the *Escherichia coli* replication terminator protein (Tus) with DNA: a model derived from DNA-binding studies of mutant proteins by surface plasmon resonance. Biochemistry 39, 11989–11999. doi: 10.1021/bi001174w, PMID: 11009613

[ref125] Neylon C. Kralicek A. V. Hill T. M. Dixon N. E. (2005). Replication termination in *Escherichia coli*: structure and antihelicase activity of the Tus-Ter complex. Microbiol. Mol. Biol. Rev. MMBR 69, 501–526. doi: 10.1128/MMBR.69.3.501-526.2005 16148308 PMC1197808

[ref126] Pai K. S. Bussiere D. E. Wang F. Hutchison C. A. 3rd White S. W. Bastia D. (1996). The structure and function of the replication terminator protein of *Bacillus subtilis*: identification of the ‘winged helix’ DNA-binding domain. EMBO J. 15, 3164–3173. doi: 10.1002/j.1460-2075.1996.tb00679.x 8670817 PMC450259

[ref127] Pandey M. Elshenawy M. M. Jergic S. Takahashi M. Dixon N. E. Hamdan S. M.. (2015). Two mechanisms coordinate replication termination by the *Escherichia coli* Tus-Ter complex. Nucleic Acids Res. 43, 5924–5935. doi: 10.1093/nar/gkv527, PMID: 26007657 PMC4499146

[ref128] Pham T. M. Tan K. W. Sakumura Y. Okumura K. Maki H. Akiyama M. T. (2013). A single-molecule approach to DNA replication in *Escherichia coli* cells demonstrated that DNA polymerase III is a major determinant of fork speed. Mol. Microbiol. 90, 584–596. doi: 10.1111/mmi.12386 23998701

[ref129] Prescott D. M. Kuempel P. L. (1972). Bidirectional replication of the chromosome in *Escherichia coli*. Proc. Natl. Acad. Sci. U. S. A. 69, 2842–2845. doi: 10.1073/pnas.69.10.2842 4562743 PMC389658

[ref130] Raghunathan N. Goswami S. Leela J. K. Pandiyan A. Gowrishankar J. (2019). A new role for *Escherichia coli* dam DNA methylase in prevention of aberrant chromosomal replication. Nucleic Acids Res. 47, 5698–5711. doi: 10.1093/nar/gkz242 30957852 PMC6582345

[ref131] Rakowski S. A. Filutowicz M. (2013). Plasmid R6K replication control. Plasmid 69, 231–242. doi: 10.1016/j.plasmid.2013.02.003 23474464 PMC3691012

[ref132] Rasko D. A. Rosovitz M. J. Myers G. S. A. Mongodin E. F. Fricke W. F. Gajer P.. (2008). The pangenome structure of *Escherichia coli*: comparative genomic analysis of *E. coli* commensal and pathogenic isolates. J. Bacteriol. 190, 6881–6893. doi: 10.1128/JB.00619-08 18676672 PMC2566221

[ref133] Rothstein R. Michel B. Gangloff S. (2000). Replication fork pausing and recombination or “gimme a break”. Genes Dev. 14, 1–10. doi: 10.1101/gad.14.1.1 10640269

[ref134] Rudolph C. J. Corocher T.-A. Grainge I. Duggin I. G. (2019). Termination of DNA replication in prokaryotes, Chichester: John Wiley & Sons, Ltd, 1–15

[ref135] Rudolph C. J. Dhillon P. Moore T. Lloyd R. G. (2007). Avoiding and resolving conflicts between DNA replication and transcription. DNA Repair 6, 981–993. doi: 10.1016/j.dnarep.2007.02.017, PMID: 17400034

[ref136] Rudolph C. J. Mahdi A. A. Upton A. L. Lloyd R. G. (2010). RecG protein and single-strand DNA exonucleases avoid cell lethality associated with PriA helicase activity in *Escherichia coli*. Genetics 186, 473–492. doi: 10.1534/genetics.110.120691 20647503 PMC2954477

[ref137] Rudolph C. J. Upton A. L. Lloyd R. G. (2009). Replication fork collisions cause pathological chromosomal amplification in cells lacking RecG DNA translocase. Mol. Microbiol. 74, 940–955. doi: 10.1111/j.1365-2958.2009.06909.x 19818016 PMC2788051

[ref138] Rudolph C. J. Upton A. L. Stockum A. Nieduszynski C. A. Lloyd R. G. (2013). Avoiding chromosome pathology when replication forks collide. Nature 500, 608–611. doi: 10.1038/nature12312 23892781 PMC3819906

[ref139] Schneiker S. Perlova O. Kaiser O. Gerth K. Alici A. Altmeyer M. O.. (2007). Complete genome sequence of the myxobacterium *Sorangium cellulosum*. Nat. Biotechnol. 25, 1281–1289. doi: 10.1038/nbt1354 17965706

[ref140] Schroeder J. W. Hirst W. G. Szewczyk G. A. Simmons L. A. (2016). The effect of local sequence context on mutational bias of genes encoded on the leading and lagging strands. Curr. Biol. CB 26, 692–697. doi: 10.1016/j.cub.2016.01.016 26923786 PMC4783269

[ref141] Schroeder J. W. Sankar T. S. Wang J. D. Simmons L. A. (2020). The roles of replication-transcription conflict in mutagenesis and evolution of genome organization. PLoS Genet. 16:e1008987. doi: 10.1371/journal.pgen.1008987 32853297 PMC7451550

[ref142] Sharma B. Hill T. M. (1992). TerF, the sixth identified replication arrest site in *Escherichia coli*, is located within the rcsC gene. J. Bacteriol. 174, 7854–7858. 1447156 10.1128/jb.174.23.7854-7858.1992 PMC207506

[ref143] Sherratt D. J. Arciszewska L. K. Crozat E. Graham J. E. Grainge I. (2010). The *Escherichia coli* DNA translocase FtsK. Biochem. Soc. Trans. 38, 395–398. doi: 10.1042/BST0380395, PMID: 20298190

[ref144] Shima N. Pederson K. D. (2017). Dormant origins as a built-in safeguard in eukaryotic DNA replication against genome instability and disease development. DNA Repair 56, 166–173. doi: 10.1016/j.dnarep.2017.06.019, PMID: 28641940 PMC5547906

[ref145] Singleton M. R. Scaife S. Wigley D. B. (2001). Structural analysis of DNA replication fork reversal by RecG. Cells 107, 79–89. doi: 10.1016/s0092-8674(01)00501-3 11595187

[ref146] Sinha A. K. Durand A. Desfontaines J.-M. Iurchenko I. Auger H. Leach D. R. F.. (2017). Division-induced DNA double strand breaks in the chromosome terminus region of *Escherichia coli* lacking RecBCD DNA repair enzyme. PLoS Genet. 13:e1006895. doi: 10.1371/journal.pgen.1006895 28968392 PMC5638614

[ref147] Sinha A. K. Possoz C. Durand A. Desfontaines J.-M. Barre F.-X. Leach D. R. F.. (2018). Broken replication forks trigger heritable DNA breaks in the terminus of a circular chromosome. PLoS Genet. 14:e1007256. doi: 10.1371/journal.pgen.1007256 29522563 PMC5862497

[ref148] Skarstad K. Katayama T. (2013). Regulating DNA replication in bacteria. Cold Spring Harb. Perspect. Biol. 5:a012922. doi: 10.1101/cshperspect.a012922 23471435 PMC3683904

[ref149] Skovgaard O. Bak M. Løbner-Olesen A. Tommerup N. (2011). Genome-wide detection of chromosomal rearrangements, indels, and mutations in circular chromosomes by short read sequencing. Genome Res. 21, 1388–1393. doi: 10.1101/gr.117416.110 21555365 PMC3149504

[ref150] Smith M. T. Wake R. G. (1992). Definition and polarity of action of DNA replication terminators in *Bacillus subtilis*. J. Mol. Biol. 227, 648–657. doi: 10.1016/0022-2836(92)90214-5 1404381

[ref151] Sonneville R. Moreno S. P. Knebel A. Johnson C. Hastie C. J. Gartner A.. (2017). CUL-2LRR-1 and UBXN-3 drive replisome disassembly during DNA replication termination and mitosis. Nat. Cell Biol. 19, 468–479. doi: 10.1038/ncb3500, PMID: 28368371 PMC5410169

[ref152] Srivatsan A. Tehranchi A. MacAlpine D. M. Wang J. D. (2010). Co-orientation of replication and transcription preserves genome integrity. PLoS Genet. 6:e1000810. doi: 10.1371/journal.pgen.1000810 20090829 PMC2797598

[ref153] Steinacher R. Osman F. Dalgaard J. Z. Lorenz A. Whitby M. C. (2012). The DNA helicase Pfh1 promotes fork merging at replication termination sites to ensure genome stability. Genes Dev. 26, 594–602. doi: 10.1101/gad.184663.111 22426535 PMC3315120

[ref154] Stoppe N. C. Silva J. S. Carlos C. Sato M. I. Z. Saraiva A. M. Ottoboni L. M. M.. (2017). Worldwide phylogenetic group patterns of *Escherichia coli* from commensal human and wastewater treatment plant isolates. Front. Microbiol. 8:2512. doi: 10.3389/fmicb.2017.02512 29312213 PMC5742620

[ref155] Syeda A. H. Dimude J. U. Skovgaard O. Rudolph C. J. (2020). Too much of a good thing: how ectopic DNA replication affects bacterial replication dynamics. Front. Microbiol. 11:534. doi: 10.3389/fmicb.2020.00534 32351461 PMC7174701

[ref156] Tanaka T. Masai H. (2006). Stabilization of a stalled replication fork by concerted actions of two helicases. J. Biol. Chem. 281, 3484–3493. doi: 10.1074/jbc.M510979200, PMID: 16354656

[ref157] Toft C. J. Moreau M. J. J. Perutka J. Mandapati S. Enyeart P. Sorenson A. E.. (2021). Delineation of the ancestral Tus-dependent replication fork trap. Int. J. Mol. Sci. 22:13533. doi: 10.3390/ijms222413533 34948327 PMC8707476

[ref158] Toft C. J. Sorenson A. E. Schaeffer P. M. (2022). A soft Tus-Ter interaction is hiding a fail-safe lock in the replication fork trap of *Dickeya paradisiaca*. Microbiol. Res. 263:127147. doi: 10.1016/j.micres.2022.127147 35914414

[ref159] Tomasetti C. Li L. Vogelstein B. (2017). Stem cell divisions, somatic mutations, cancer etiology, and cancer prevention. Science 355, 1330–1334. doi: 10.1126/science.aaf9011 28336671 PMC5852673

[ref160] Touchon M. Rocha E. P. C. (2016). Coevolution of the organization and structure of prokaryotic genomes. Cold Spring Harb. Perspect. Biol. 8:a018168. doi: 10.1101/cshperspect.a018168 26729648 PMC4691797

[ref161] Trautinger B. W. Jaktaji R. P. Rusakova E. Lloyd R. G. (2005). RNA polymerase modulators and DNA repair activities resolve conflicts between DNA replication and transcription. Mol. Cell 19, 247–258. doi: 10.1016/j.molcel.2005.06.004 16039593

[ref162] Wake R. G. (1972). Visualization of reinitiated chromosomes in *Bacillus subtilis*. J. Mol. Biol. 68, 501–509. doi: 10.1016/0022-2836(72)90102-7 4627106

[ref163] Wake R. G. (1973). Circularity of the *Bacillus subtilis* chromosome and further studies on its bidirectional replication. J. Mol. Biol. 77, 569–575. doi: 10.1016/0022-2836(73)90223-4, PMID: 4198889

[ref164] Wake R. G. (1997). Replication fork arrest and termination of chromosome replication in *Bacillus subtilis*. FEMS Microbiol. Lett. 153, 247–254. doi: 10.1111/j.1574-6968.1997.tb12581.x 9271849

[ref165] Wang J. D. Berkmen M. B. Grossman A. D. (2007). Genome-wide coorientation of replication and transcription reduces adverse effects on replication in *Bacillus subtilis*. Proc. Natl. Acad. Sci. U. S. A. 104, 5608–5613. doi: 10.1073/pnas.0608999104 17372224 PMC1838449

[ref166] Wang X. Lesterlin C. Reyes-Lamothe R. Ball G. Sherratt D. J. (2011). Replication and segregation of an *Escherichia coli* chromosome with two replication origins. Proc. Natl. Acad. Sci. U. S. A. 108, E243–E250. doi: 10.1073/pnas.1100874108, PMID: 21670292 PMC3127894

[ref167] Weiss A. S. Hariharan I. K. Wake R. G. (1981). Analysis of the terminus region of the *Bacillus subtilis* chromosome. Nature 293, 673–675. doi: 10.1038/293673a0 6270570

[ref168] Wendel B. M. Cole J. M. Courcelle C. T. Courcelle J. (2018). SbcC-SbcD and ExoI process convergent forks to complete chromosome replication. Proc. Natl. Acad. Sci. U. S. A. 115, 349–354. doi: 10.1073/pnas.1715960114 29208713 PMC5777064

[ref169] Wendel B. M. Courcelle C. T. Courcelle J. (2014). Completion of DNA replication in *Escherichia coli*. Proc. Natl. Acad. Sci. U. S. A. 111, 16454–16459. doi: 10.1073/pnas.1415025111, PMID: 25368150 PMC4246274

[ref170] Wilce J. A. Vivian J. P. Hastings A. F. Otting G. Folmer R. H. A. Duggin I. G.. (2001). Structure of the RTP–DNA complex and the mechanism of polar replication fork arrest. Nat. Struct. Biol. 8, 206–210. doi: 10.1038/84934 11224562

[ref171] Winterstein C. Ludwig B. (1998). Genes coding for respiratory complexes map on all three chromosomes of the *Paracoccus denitrificans* genome. Arch. Microbiol. 169, 275–281. doi: 10.1007/s002030050572 9531627

[ref172] Xie B.-B. Rong J.-C. Tang B.-L. Wang S. Liu G. Qin Q.-L.. (2021). Evolutionary trajectory of the replication mode of bacterial replicons. mBio 12:e02745-20. doi: 10.1128/mBio.02745-20, PMID: 33500342 PMC7858055

[ref173] Xu Z.-Q. Dixon N. E. (2018). Bacterial replisomes. Curr. Opin. Struct. Biol. 53, 159–168. doi: 10.1016/j.sbi.2018.09.006 30292863

[ref174] Zhou H. Zaher M. S. Walter J. C. Brown A. (2021). Structure of CRL2Lrr1, the E3 ubiquitin ligase that promotes DNA replication termination in vertebrates. Nucleic Acids Res. 49, 13194–13206. doi: 10.1093/nar/gkab1174, PMID: 34850944 PMC8682755

